# Massive and massless plasmons in germanene nanosheets

**DOI:** 10.1038/s41598-022-23058-3

**Published:** 2022-11-03

**Authors:** Michele Pisarra, Cristian Vacacela Gomez, Antonello Sindona

**Affiliations:** 1 Gruppo Collegato di Cosenza, Sezione dei Laboratori Nazionali di Frascati (LNF), Istituto Nazionale di Fisica Nucleare (INFN), Cubo 31C, 87036 Rende, CS Italy; 2 Facultad de Ciencias, Escuela Superior Politécnica de Chimborazo (ESPOCH), Riobamba, 060155 Ecuador; 3 Dipartimento di Fisica, Università della Calabria, Via P. Bucci, Cubo 30C, 87036 Rende, CS Italy

**Keywords:** Electronic properties and materials, Graphene, Optics and photonics, Polaritons, Graphene, Electronic properties and devices, Optical properties and devices

## Abstract

Atomically thin crystals may exhibit peculiar dispersive electronic states equivalent to free charged particles of ultralight to ultraheavy masses. A rare coexistence of linear and parabolic dispersions yields correlated charge density modes exploitable for nanometric light confinement. Here, we use a time-dependent density-functional approach, under several levels of increasing accuracy, from the random-phase approximation to the Bethe-Salpeter equation formalism, to assess the role of different synthesized germanene samples as platforms for these plasmon excitations. In particular, we establish that both freestanding and some supported germenene monolayers can sustain infrared massless modes, resolved into an out-of-phase (optical) and an in-phase (acoustic) component. We further indicate precise experimental geometries that naturally host infrared massive modes, involving two different families of parabolic charge carriers. We thus show that the interplay of the massless and massive plasmons can be finetuned by applied extrinsic conditions or geometry deformations, which constitutes the core mechanism of germanene-based optoelectronic and plasmonic applications.

## Introduction

The understanding of collective electron phenomena at the nanoscale is a main theme in the research on novel artificial heterostructures and related device architectures^1–4^. In this exploration, a number of superior qualities are offered by two-dimensional (2D) crystals with linear electronic bands, or Dirac cones, crossing around the Fermi energy ${E_\mathrm{{F}}}$.

Such 2D Dirac cone materials (2DDMs) host massless charge carriers of large group velocities^5,6^, which strongly couple to light via excited charge density waves quantized as *plasmons*^7–9^. The Dirac cones with vertices at ${E_\mathrm{{F}}}$ are commonly characterized at the corners (K points) of the first Brillouin zone (1$^\mathrm{st}$BZ) in graphene, the first isolated 2DDM composed of carbon atoms in a honeycomb lattice^10–13^.

However, in spite of the hundreds of 2D materials discovered so far, experimental observations of 2DDMs other than graphene are rather few^14–28^. In particular, a number of single and multiple low-buckled hexagonal phases of germanium were characterized on gold^15^, aluminum^16,17^, and silver^18,19^. Some alternative realizations of this kind, grown on large to moderate gapped substrates, such as AlN^23^ and $\mathrm{{MoS}}_{2}$^24,25^, showed clear hallmarks Dirac cones near ${E_\mathrm{{F}}}$. The corresponding overlayers, here referred to as *quasifreestanding* germanene (QFGe) sheets, additionally exhibited another bunch of parabolically dispersing electronic states approaching or even crossing $E_\mathrm{{F}}$.

These achievements, while confirming the massless nature of the charge carriers, derived from the linear bands, also indicate coexistence of massive charge carriers, originating from the parabolic bands, which further boosts the interest in germanene-based 2DDMs. Similar features were observed in metal quantum well structures grown on graphene^29^, while a more extreme correlation of linear and flat bands was recognized in twisted sandwiched graphene^30^.

Given these premises, a major issue is on the dielectric response and related plasmon modes of the QFGe sheets, as compared to freestanding germanene (FGe). In this respect, particular attention sholud be given to the role played by plasmons in extreme light trapping.

Here we provide such a study, starting from a time-dependent density-functional theory^31–34^ (TDDFT) approach, in the random phase approximation (RPA), with a local kernel designed for 2D systems^35–44^. Accordingly, we compute the optical absorption and energy loss function of the FGe and QFGe monolayers that allow us to explore their leading single-particle excitations (SPEs) processes and charge density modes, over the infrared (IR) to the ultraviolet (UV) range.

Next, we consider the explicit inclusion of quasiparticle GW corrections^45–50^, in an RPA+GW approach. Finally, we compare our results with optical macroscopic permittivity calculations, performed within the Bethe-Salpeter equation (BSE) and BSE+GW frameworks^51–54^.

We demonstrate the existence of tunable massless and massive plasmons as a unique manifestation of strongly interacting 2D quantum matter, providing at the same time careful control tools to monitor their correlated propagation and damping.

## Results

### Band dispersions and density of states

We carried out the electronic structure calculations using the plane-wave (PW) approach^57,58^ to Kohn–Sham (KS) density-functional-theory (DFT), within the local density approximation^59,60^ (LDA) supported by an efficient norm-conserving pseudopotential^61^.

**Figure 1:**
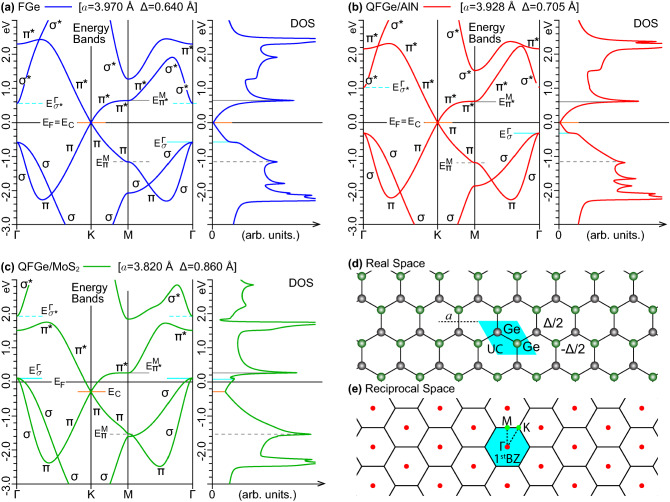
Band structure and DOS profiles of (**a**) FGe, (**b**) $\mathrm{{QFGe{\,}on{\,}AlN}}$, and (**c**) $\mathrm{{QFGe{\,}on{\,}MoS}}_{2}$, within an energy window of 6 eV centered at the Fermi level (${E_\mathrm{{F}}}{=}0$). The energy dispersions are associated to occupied or empty states with dominant ${\sigma }$ (${\sigma ^{*}}$) and ${\pi }$ (${\pi ^{*}}$) characters. The DOS curves are shown convolved with a Lorentzian lineshape having a phenomenological broadening of 0.01 eV. The other energy labels, and related horizontal lines, denote the positions of the Dirac cone ($E_\mathrm{{C}}$), the highest (degenerate) $\sigma$-like states at ${\Gamma }$ ($E_{\sigma }^{\Gamma }$), the lowest $\sigma ^{*}$-like states at ${\Gamma }$ ($E_{{\sigma }^{*}}^{\Gamma }$), the highest $\pi$-like VHS at M ($E^{\pi }_\mathrm{{M}}$), and the lowest $\pi ^{*}$-like VHS at M ($E^{\pi ^{*}}_\mathrm{{M}}$). (**d**) Real and (**e**) reciprocal space information, i.e., unit cell (UC), crystal basis, lattice constant *a*, buckling distance $\Delta$, 1$^\mathrm{st}$BZ and irreducible 1$^\mathrm{st}$BZ, delimited by the ${\Gamma }\mathrm{{KM}}{\Gamma }$ path, being also the horizontal axis in the left panels of (**a**)–(**c**).

Figure 1 reports the energy bands, along the high-symmetry ${\Gamma }\mathrm{{KM}}{\Gamma }$ path of the 1$^\mathrm{st}$BZ, and the density of states (DOS) of the two above outlined QFGe sheets, in comparison with FGe. The key electronic states implicated in optical processes belong to the two highest bands below and the two lowest bands above the Dirac cone vertex, at energy $E_\mathrm{{C}}$. The energy-wave-vector dispersion of these bands are highly affected by the equilibrium geometry of the corresponding lattices, which also determines the peak positions and widths of the associated DOS profiles. Nonetheless, some typical trends of group IV monolayers with honeycomb lattice can be identified.

In particular, the Dirac cones emerge at the K point, being characterized by two bands of dominant $\pi$ and $\pi ^{*}$ character, apparent in the projected band structures (see Supplementary Information, Sec. I). The cone shape is practically untouched by geometry effects, with a slope (or Fermi velocity) of 0.24, in units of the Bohr velocity, being about 63% of the Fermi velocity of freestanding graphene, as calculated within the LDA^43^. The $\pi$-like and $\pi ^{*}$-like bands approach the M point with flat dispersions, associated to the first van Hove singularity (VHS) pair in the DOS profiles. The second highest band, below $E_\mathrm{{C}}$, exhibits two non-degenerate minima with $\sigma$-like character towards the middle of the ${{\Gamma }\mathrm{{K}}}$ and ${\mathrm{{M}}{\Gamma }}$ lines, where the first lowest band, above $E_\mathrm{{C}}$, presents two non-degenerate maxima with $\pi ^{*}$-like character.

An unparalleled feature is the behavior of the highest occupied and lowest unoccupied states around $\Gamma$, which respectively form two $\sigma$-like bands, approaching the degenerate energy $E_{\sigma }^{\Gamma }$, and one $\sigma ^{*}$-like or $\pi ^{*}$-like band, depending on the system’s geometry. Unlike graphene and silicene^26^, the energies of the $\sigma$-like states are sufficiently close to $E_\mathrm{{F}}$ that substrate-induced lattice deformations gradually turn the nature of the germanene overlayer from *zero gap semimetal* (FGe and $\mathrm{{QFGe{\,}on{\,}AlN}}$) to *metal* ($\mathrm{{QFGe{\,}on{\,}MoS}}_{2}$).

Specifically, the AlN-induced compression (2.1%) shifts up the $\sigma$-like and $\sigma ^{*}$-like bands of the QFGe overlayer, leaving unaltered the $\pi$-like and $\pi ^{*}$-like bands, with inclusion of the Dirac cone at vertex $E_\mathrm{{C}}{=}E_\mathrm{{F}}$ and the VHSs’ structure (Fig. 1b). This QFGe system is still a semimetal, along with FGe, though the degenerate top level of the $\sigma$-like bands is increased by $0.30\,\mathrm{eV}$ towards $E_\mathrm{{F}}$, which corresponds to a higher onset of the decreasing behavior of the occupied DOS, vanishing at the Dirac cone.

The $\mathrm{{MoS}}_{2}$-induced compression (7.4%) produces a more significant upshift of the $\sigma$-like bands that cross the Fermi level at $\Gamma$, with $E_{\sigma }^{\Gamma }$ lying at ${\sim }0.11\,\mathrm{eV}$ above $E_\mathrm{{F}}$, which yields a small spike in the associated DOS (Fig. 1c). As a result, this other QFGe system is a metal with the Dirac cone downshifted to $E_\mathrm{{C}}{=}{-}0.29$ eV below $E_\mathrm{{F}}$, corresponding to a non-vanishing DOS point, and a *hole pocket* left at $\Gamma$ with two different dispersions. The latter are equivalent to two families of positive charge carriers of effective masses $0.45{\,}{m_{{ \textsc {e}}}}$ and $0.07{\,}{m_{{ \textsc {e}}}}$, with ${m_{{ \textsc {e}}}}$ denoting the electron rest mass.

Further tuning of $E_\mathrm{{F}}$ in synthesized germanene overlayers can be achieved by proper combination of chemical doping or electrostatic gating and mechanical stress or strain^62–66^.

### Dielectric properties

The above outlined electronic structures are primarily involved in the macroscopic permittivity response $\epsilon ^{{ \textsc {m}}}$ of the FGe and QFGe monolayers. The other key element is the interaction generated by light-induced changes in charge density that we approximated to a truncated Coulomb potential, specific for 2D materials^40–44^, see sections "Absorption Spectra and Loss Spectra". We used the same potential, in conjunction with a plasmon pole model^47^, to correct the band energies of Fig. 1 at the level of the GW approximation^48–50^, as reported in section  "Many-body quasiparticle and excitonic effects". We further considered other two-particle excitonic effects within the BSE approximation^52–54^, see also section "Many-body quasiparticle and excitonic effects".

In the following, we present the behavior of $\mathrm{im}(\epsilon ^{{ \textsc {m}}})$ and $-\mathrm{im}(1/\epsilon ^{{ \textsc {m}}})$ *vs* the probing energy $\omega$ and transferred momentum $\mathbf{q}$. These two quantities, being respectively proportional to the absorption cross-section and the so-called energy-loss function, provide complementary spectral representations of plasmon propagation and damping. The peak structures of $-\mathrm{im}(1/\epsilon ^{{ \textsc {m}}})$ are blueshifted relative to $\mathrm{im}(\epsilon ^{{ \textsc {m}}})$, with the plasmon resonances following the absorption peaks and lying just below the loss peaks, at the closest-to-zero permittivity point^67^. Undamped plasmons are more markedly spotted in the double sign change of $\mathrm{re}(\epsilon ^{{ \textsc {m}}})$, in particular, at the largest zero of $\mathrm{re}(\epsilon ^{{ \textsc {m}}})$, which matches the negligibly small value of $\mathrm{im}(\epsilon ^{{ \textsc {m}}})$ relative to its peak (see Supplementary Information, Sec. III).

### Charge carrier concentration

Our main concern is on doping- or gating- induced shifting of $E_\mathrm{{F}}$ at fixed (room) temperature. This amounts to inject or eject small electron concentrations ${n_\mathrm{{e}}}$ or ${n_\mathrm{{h}}}$ that cause positive or negative shifts ${\Delta }E_\mathrm{{F}}$, while leaving unaltered the underlying electronic structure of the intrinsic systems.

**Figure 2:**
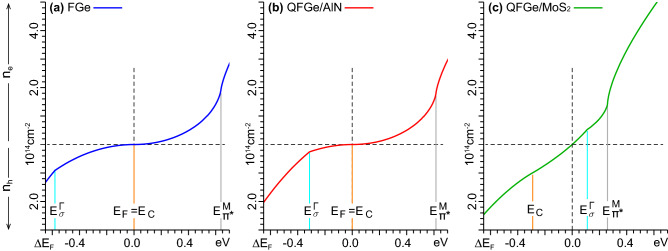
Electron (${n_\mathrm{{e}}}$) or hole (${n_\mathrm{{h}}}$) carrier concentrations *vs* ${{\Delta }E_\mathrm{{F}}}{>}0$ or ${{\Delta }E_\mathrm{{F}}}{<}0$ in (**a**) FGe, (**b**) $\mathrm{{QFGe{\,}on{\,}AlN}}$, and (**c**) $\mathrm{{QFGe{\,}on{\,}MoS}}_{2}$, with ${\Delta }E_\mathrm{{F}}{=}0$ denoting the charge neutrality point. $E_{\sigma }^{\Gamma }$, $E_\mathrm{{C}}$, and $E^{\pi ^{*}}_\mathrm{{M}}$, labeled as in Fig. 1(**a**)–(**c**), are associated to non-smooth, distinct trends of the three curves.

Figure 2 shows the ${n_\mathrm{{e/h}}}$ profiles against ${\Delta }E_\mathrm{{F}}$, within the range where the highest occupied valence band, and the lowest unoccupied conduction band, have linear ($\pi$-like or $\pi ^{*}$-like), parabolic ($\sigma$-like), and flat ($\pi ^{*}$-like) dispersions around K, $\Gamma$, and M, respectively.

In FGe, the filling or emptying of the Dirac cone states is the main mechanism for doping or gating, which activates a 2D optical plasmon (2DP) similarly to graphene and silicene^37,38^.

In $\mathrm{{QFGe{\,}on{\,}AlN}}$, a moderate shift ${\Delta }E_\mathrm{{F}}{\lesssim }{-}0.3\,\mathrm{eV}$, i.e., an ejected electron density ${n_\mathrm{{h}}}{\gtrsim }3{\times }10^{13}\,\mathrm{cm}^{-2}$, is sufficient to empty part of the $\sigma$-like bands and leave a hole pocket at $\Gamma$, which produces another $\sigma$-like plasmon ($\sigma$P) interacting with the 2DP.

In $\mathrm{{QFGe{\,}on{\,}MoS}}_{2}$, a consistent hole pocket is already present in the intrinsic system (set by ${\Delta }E_\mathrm{{F}}{=}0$ or ${n_\mathrm{{e/h}}}{=}0$), along with strongly overlapping 2DP and $\sigma$P modes. Significant variations of ${n_\mathrm{{e/h}}}$, around $10^{11}\,\mathrm{cm}^{{-}2}$, may be locally induced by point defects in $\mathrm{{MoS}}_{2}$^25^. Nonetheless, much larger injected electron densities, ${n_\mathrm{{e}}}{\gtrsim }5.5{\times }10^{13}\,\mathrm{cm}^{{-}2}$, are required to shift $E_\mathrm{{F}}$ above the $\sigma$-like bands and deactivate the $\sigma$P. Conversely, an ejected electron density ${n_\mathrm{{h}}}{=}9.8{\times }10^{13}\,\mathrm{cm}^{{-}2}$ can restore $E_\mathrm{{F}}$ at the Dirac cone vertex.

In all cases, the unoccupied VHS states act as a barrier to Fermi level shifting (Fig. 1b,c), making it hard to achieve values of ${{\Delta }E_\mathrm{{F}}}$ larger than 0.4eV in FGe or $\mathrm{{QFGe{\,}on{\,}AlN}}$, and 0.25eV in $\mathrm{{QFGe{\,}on{\,}MoS}}_{2}$.

### Absorption spectra

We begin by analyzing the absorption properties of the germanene monolayers in their intrinsic state, at the the level of TDDFT in our RPA approach. Accordingly, we focus on the energy dependence of the macroscopic imaginary permittivity ${\mathrm{im}}(\epsilon ^{{ \textsc {m}}})$ in the optical momentum limit, i.e., at a fixed momentum transfer of 2.5-$2.6{\times }10^{-3}\,\mathrm{\text{\AA} }^{-1}$, which corresponds to the typical momentum of a photon in the few-eV energy range.

**Figure 3:**
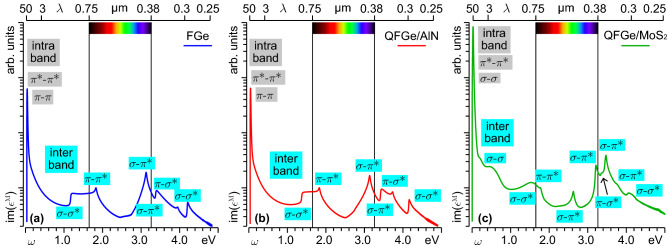
Intrinsic absorption spectra [${\propto }\mathrm{im}(\epsilon ^{{ \textsc {m}}})$] of (**a**) FGe, (**b**) $\mathrm{{QFGe{\,}on{\,}AlN}}$, and (**c**) $\mathrm{{QFGe{\,}on{\,}MoS}}_{2}$ in the energy range ${\omega }{<}5$ eV, or wavelength range ${\lambda }{\ge }250$nm. The same vertical logarithmic scale in arbitrary (arb.) units is adopted for the three curves. Intraband SPEs (gray-box labels) involve $\pi$-like, $\pi ^{*}$-like, and $\sigma$-like states around ${E_\mathrm{{F}}}$, yielding the sharp FIR-MIR peak. Interband SPEs (cyan-box labels) result from transitions from the $\sigma$-like and $\pi$-like, below ${E_\mathrm{{F}}}$, to the $\sigma$-like, $\sigma ^{*}$-like and $\pi ^{*}$-like states, above ${E_\mathrm{{F}}}$, producing the NIR-VIS and VIS-MUV peak structures in (**a**)–(**c**), plus the MIR-NIR shoulder in (**c**).

Three main peak structures are distinguished in the far-infrared (FIR) to mid-infrared (MIR), near-infrared (NIR) to visible (VIS), and VIS to mid-ultraviolet (MUV) regimes, as attested by the absorption lineshapes of Fig. 3.

The sharp FIR-MIR peak, at ${\sim }0.02\,\mathrm{eV}$, is associated to intraband single particle excitations (SPEs) around the Fermi level. In FGe and $\mathrm{{QFGe{\,}on{\,}AlN}}$, this feature results from quasivertical transitions occurring at the K point of the 1$^\mathrm{st}$BZ, thus involving thermally excited $\pi$-like and $\pi ^{*}$-like charge carriers at the Dirac cone, which is a consequence of the semimetal nature of the monolayers. A higher peak intensity, of about a factor of eight, is recorded in $\mathrm{{QFGe{\,}on{\,}MoS}}_{2}$, because of the Fermi level shifting above the Dirac cone, and the corresponding metal nature of the system. By inspecting the differences in the charge-carrier concentration profiles of Fig. 2, we have estimated a population of ${n_\mathrm{{e}K}}=2.4{\times }10^{13}\,\mathrm{cm}^{{-}2}$ electrons in the $\pi ^{*}$-like part of the Dirac cone, between ${E_\mathrm{{C}}}$ and ${E_\mathrm{{F}}}$. We have further estimated a population of ${n_\mathrm{{h}\Gamma }}=2.8{\times }10^{13}\,\mathrm{cm}^{{-}2}$ holes in the $\sigma$-like bands, between ${E_\mathrm{{F}}}$ and ${E_{\sigma }^{\Gamma }}$. Accordingly, the FIR-MIR peak in $\mathrm{{QFGe{\,}on{\,}MoS}}_{2}$ is determined by intraband SPEs around K and $\Gamma$.

A shoulder appears in $\mathrm{{QFGe{\,}on{\,}MoS}}_{2}$ at MIR to NIR wavelengths, i.e., in the energy range between 0.4 and 0.8eV. This is a signature of interband SPEs between the occupied and empty $\sigma$-like states at the crossing point with ${E_\mathrm{{F}}}$, as confirmed by a joint DOS analysis (see Supplementary Information, Sec. II). These $\sigma$-$\sigma$ excitations originate from parabolic-like bands and contribute with a broader and much less intense peak, being partly superimposed with the MIR tail of the FIR-MIR peak.

The NIR to VIS peak structure extends from 1.2eV in FGe, 1.4eV in $\mathrm{{QFGe{\,}on{\,}AlN}}$, and 1.5eV in $\mathrm{{QFGe{\,}on{\,}MoS}}_{2}$, to 1.8eV, being mostly determined by interband SPEs around $\Gamma$ and M (see Supplementary Information, Sec. II).

In FGe and $\mathrm{{QFGe{\,}on{\,}AlN}}$ the onset energy, on the NIR range, coincides with the gap between the $\sigma$-like and $\sigma ^{*}$-like bands at $\Gamma$, thus involving $\sigma$-$\sigma ^{*}$ SPEs between the corresponding band maxima and minima.

In $\mathrm{{QFGe{\,}on{\,}MoS}}_{2}$, the initial NIR structure has a smoother trend, originating from transitions from the occupied $\sigma$-like states to the unoccupied $\sigma ^{*}$-like states at $\Gamma$. Accordingly, the $\sigma$-$\sigma ^{*}$ SPEs lead to a broad maximum around 1.6eV, due to transitions to the $\sigma ^{*}$ band minimum.

In all monolayers the VIS peak at 1.8eV arises from transitions from the $\pi$-like to $\pi ^{*}$-like bands, at the corresponding VHS points. The latter appears as a weak feature in $\mathrm{{QFGe{\,}on{\,}MoS}}_{2}$, being superimposed to the spectrum of $\sigma$-$\sigma ^{*}$ SPEs, away from $\Gamma$.

The VIS to MUV peak structure, covering the 2.6-$4.6\,\mathrm{eV}$ range, involves SPEs between the highest (or second-highest) valence band and the second-lowest (or lowest) conduction band, with dominant transitions from $\pi$-like (or $\sigma$-like) to $\sigma ^{*}$-like (or $\pi ^{*}$-like) states, around the corresponding DOS peaks (see Supplementary Information, Sec. II).

In FGe and $\mathrm{{QFGe{\,}on{\,}AlN}}$, the 3.1 and 3.3–3.4 eV peaks mainly originate from $\sigma$-$\pi ^{*}$ SPEs, with a maximum intensity due to transitions towards $\Gamma$, and around the mid points the ${{\Gamma }\mathrm{{K}}}$ and ${\mathrm{{M}}{\Gamma }}$ segments. The weak 3.7-3.9 eV peak is mostly due to $\pi$-$\sigma ^{*}$ SPEs around the mid points of the ${\mathrm{{K}}\mathrm{{M}}}$ and ${\mathrm{{M}}{\Gamma }}$ lines. The 4.1-4.2 eV peak has a main contribution from $\sigma$-$\sigma ^{*}$ SPEs along the ${{\Gamma }\mathrm{{K}}}$ and ${\mathrm{{M}}{\Gamma }}$ lines, close to $\Gamma$.

In $\mathrm{{QFGe{\,}on{\,}MoS}}_{2}$, all the 2.6, 3.2, 3.5, and 4.1 eV peaks mainly originate from $\sigma$-$\pi ^{*}$ SPEs around the mid points of the ${{\Gamma }\mathrm{{K}}}$ and ${\mathrm{{M}}{\Gamma }}$ segments. Other contributions at 3.4 eV and 3.5 eV are respectively due to $\pi$-$\sigma ^{*}$ SPEs around M and $\sigma$-$\sigma ^{*}$ SPEs along ${{\Gamma }\mathrm{{K}}}$, close to $\Gamma$.

The different peak positions and intensities of the VIS to MUV feature in $\mathrm{{QFGe{\,}on{\,}MoS}}_{2}$, as compared to FGe and $\mathrm{{QFGe{\,}on{\,}AlN}}$, are a consequence of the different positions of the DOS peaks around the $\pi$-like (or $\sigma$-like) band minima and the $\sigma ^{*}$-like (or $\pi ^{*}$-like) band maxima.

### Loss spectra

**Figure 4:**
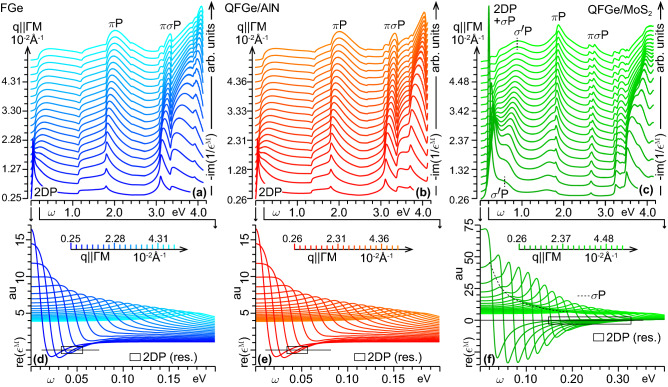
(Top) Intrinsic loss functions [${\propto }{-}\mathrm{im}(1/\epsilon ^{{ \textsc {m}}})$, in arb. units] of (**a**) FGe, (**b**) $\mathrm{{QFGe{\,}on{\,}AlN}}$, and (**c**) $\mathrm{{QFGe{\,}on{\,}MoS}}_{2}$ displayed as vertically shifted plots *vs* ${\omega }{<}4.2$ eV, for some fixed sampled momenta ${q}{<}5.4{\times }10^{-2}\,\mathrm{\text{\AA} }^{-1}$ with ${\mathbf{q}}{\parallel }{{\Gamma }\mathrm{{M}}}$. The slight differences in the *q*-values of the three monolayers are a consequence of their different lattice constants (see Fig. 1). The ${\omega }{<}0.4$ eV dispersive features originate from intraband charge density oscillations, namely the 2DP mode in (**a**), (**b**), and the 2DP+$\sigma$P modes in (**c**), with the highest peak of FGe and $\mathrm{{QFGe{\,}on{\,}AlN}}$, at ${\sim }0.02$eV, being 20% lower than that of $\mathrm{{QFGe{\,}on{\,}MoS}}_{2}$, at ${\sim }0.04$ eV. The ${\omega }{>}0.4$ eV dispersive peaks exhibit similar intensities at similar momentum transfers, being a signature of different interband plasmons, including the ${\sigma ^{\prime }}P$ mode [dashed lines in (**c**)], plus the more conventional ${\pi }P$ and ${\pi \sigma }P$ modes in (**a**)–(**c**). (Bottom) Real macroscopic permittivity [${\mathrm{re}}(\epsilon ^{{ \textsc {m}}})$, in atomic units (au)] of (**d**) FGe, (**e**) $\mathrm{{QFGe{\,}on{\,}AlN}}$, *vs* ${\omega }{<}0.2$ eV, and (**f**) $\mathrm{{QFGe{\,}on{\,}MoS}}_{2}$ *vs* ${\omega }{<}0.4$ eV, with the same sampled momenta of (**a**)–(**c**). The black boxes highlight the undamped 2DP resonances, and the dashed line follows the effect of the damped $\sigma$P.

We now move to the energy loss properties of the three monolayers, within the TDDFT-RPA framework. As a preliminary analysis, we focus on the behavior of ${-}\mathrm{im}(1/\epsilon ^{{ \textsc {m}}})$ at small momentum transfers along ${\Gamma }\mathrm{{M}}$, say, ${q}{<}5.4{\times }10^{-2}\,\mathrm{\text{\AA} }^{-1}$ down to the optical limit, displayed in Fig. 4. We notice the existence of distinct dispersive peak structures, which at the lowest sampled momentum correspond to the FIR-MIR, MIR-NIR, NIR-VIS and VIS-MUV absorption features of Fig. 3.

In particular, FGe and $\mathrm{{QFGe{\,}on{\,}AlN}}$ exhibit a 2DP mode of low intensity at 0.01-0.04 eV, due to charge density oscillations of the fraction of thermally excited Dirac cone electrons and holes at room temperature, with the 2DP peaks in Fig. 4a,b representing the loss counterpart of the FIR-MIR absorption peaks in Fig. 3a,b.

In $\mathrm{{QFGe{\,}on{\,}MoS}}_{2}$, on the other hand, we detect an intriguing scenario where the $0.29\,\mathrm{eV}$ shifting of $E_\mathrm{{F}}$ above $E_\mathrm{{C}}$ induces a strong 2DP at 0.04-0.3eV, involving the above-calculated ${n_\mathrm{{e}K}}$ concentration of massless Dirac cone electrons. This oscillation coexists with the intraband modes ($\sigma$P) of the above-calculated ${n_\mathrm{{h}\Gamma }}$ concentration of massive parabolic holes, associated with the two $\sigma$-like bands, which cross the Fermi energy close to $\Gamma$. The massless and massive modes are superimposed to each other, and cannot be disentangled in intrinsic conditions. This yields the 2DP+$\sigma$P peak in Fig. 4b, which corresponds to the FIR-MIR absorption peak of Fig. 3c. The peak to peak ratio of the 2DP, in FGe and $\mathrm{{QFGe{\,}on{\,}AlN}}$, and the 2DP+$\sigma$P, in $\mathrm{{QFGe{\,}on{\,}MoS}}_{2}$, at the lowest sampled momentum, parallels the difference in the absorption peak intensities displayed in Fig. 3.

Looking at the real macroscopic permittivity, in Fig. 4d–f, we can further observe that the 2DP propagates undamped in the Landau sense^55,56^ over a different momentum transfer range, namely, $q{<}0.8{\times }10^{-2}\,\mathrm{\text{\AA} }^{-1}$, in FGe and $\mathrm{{QFGe{\,}on{\,}AlN}}$, and $q{<}3.2{\times }10^{-2}\,\mathrm{\text{\AA} }^{-1}$, in $\mathrm{{QFGe{\,}on{\,}MoS}}_{2}$, where $\mathrm{re}(\epsilon ^{{ \textsc {m}}})$ presents a well-defined pair of zeros. On the other hand, the $\sigma$P modes of $\mathrm{{QFGe{\,}on{\,}MoS}}_{2}$ are largely damped, being not related to a clear change of sign in $\mathrm{re}(\epsilon ^{{ \textsc {m}}})$, though they leave a signature in $\mathrm{re}(\epsilon ^{{ \textsc {m}}})$ below the 2DP zeros.

Another peculiar collective motion of the $\sigma$-like charge carriers in $\mathrm{{QFGe{\,}on{\,}MoS}}_{2}$ is related to the 0.4-1.3eV dispersive feature in Fig. 4c, whose lowest momentum peak at ${\sim }0.6\,\mathrm{eV}$ corresponds to the MIR shoulder in the absorption spectrum of Fig. 3c. The sequence of peak positions, ranging from 0.6 to 0.9 eV with increasing *q*, suggests that this feature is a manifestation of the interband plasmon ($\sigma ^{\prime }$P) assisted by SPEs between the occupied and empty metal states at $\Gamma$.

The other two peak structures, at 1.4-2.2eV and 2.6-5.0eV, are the optical counterparts of the so-called $\pi$-like plasmon ($\pi$P) and $\pi \sigma$-like plasmon ($\pi \sigma$P), which are commonly characterized in group IV honeycomb sheets and heterostructures. These appear as largely damped charge density oscillations, being respectively superimposed to the $\pi$-$\pi ^{*}$ SPE spectrum and the $\pi$-$\sigma ^{*}$, $\sigma$-$\sigma ^{*}$, $\sigma$-$\pi ^{*}$ SPE spectra.

**Figure 5:**
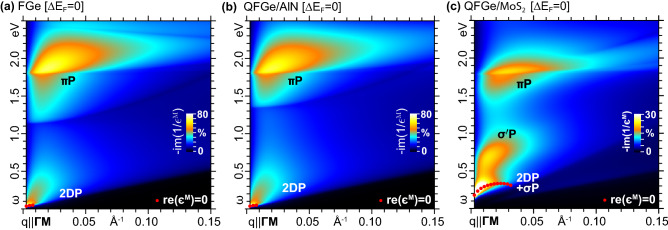
Intrinsic loss functions (in arb. units) of (**a**) FGe, (**b**) $\mathrm{{QFGe{\,}on{\,}AlN}}$ and (**c**) $\mathrm{{QFGe{\,}on{\,}MoS}}_{2}$, displayed as density maps *vs* $\omega {<}2.5$eV and ${q}{<}0.15\,\mathrm{\text{\AA} }^{-1}$ with ${\mathbf{q}}{\parallel }{{\Gamma }\mathrm{{M}}}$. The color scale is normalized to the maximum intensity of the $\omega {<}0.4$ eV mode, with the red dots labeling the undamped plasmon resonances. The dominant intraband (2DP, $\sigma$P) and interband ($\sigma ^{\prime }$P, $\pi$P) modes are highlighted as in Fig. 3.

An even more informative representation is provided by the density maps of the FIR to VIS modes, given in Fig. 5 for a broad range of momentum transfers ${q}{<}0.15\,\mathrm{\text{\AA} }^{-1}$ along ${{\Gamma }\mathrm{{M}}}$. FGe (Fig. 5a) and $\mathrm{{QFGe{\,}on{\,}AlN}}$ (Fig. 5b) present very similar loss spectra, sharing an identical weak 2DP mode with monotonically increasing dispersive trend. An appreciable difference is detectable in the onset of the $\pi$P structure, based on the positions of the top ($\sigma$-like) and bottom ($\sigma ^{*}$-like) band levels at $\Gamma$, as discussed above with reference to Figs. 1a,b and 2a,b.

Again, the most interesting feature is due to the 2DP+$\sigma$P and $\sigma ^{\prime }$P modes in $\mathrm{{QFGe{\,}on{\,}MoS}}_{2}$ (Fig. 5c), which follow an interfering pathway due to the different massless and massive plasmons involved. Also visible in Fig. 5a–c is that the $\pi$P peak position increases monotonically with increasing *q*, though the actual dispersion, width and onset of the associated spectral structures in FGe and $\mathrm{{QFGe{\,}on{\,}AlN}}$ *vs* $\mathrm{{QFGe{\,}on{\,}MoS}}_{2}$ are significantly different, as a consequence of the semimetal *vs* metal nature of the monolayers.

**Figure 6:**
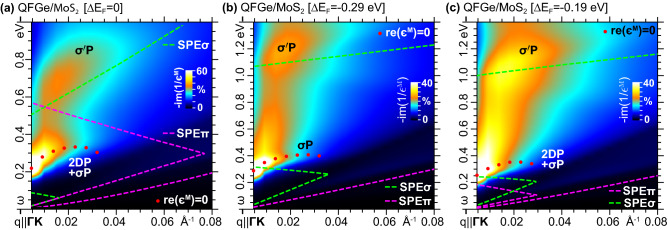
Loss functions (in arb. units) of $\mathrm{{QFGe{\,}on{\,}MoS}}_{2}$, displayed as density maps *vs* $\omega {<}1.4$ eV and ${q}{<}0.08\,\mathrm{\text{\AA} }^{-1}$, with ${\mathbf{q}}{\parallel }{{\Gamma }\mathrm{{K}}}$, for different doping or gating conditions, with the Fermi level driven from the intrinsic state (**a**) to the Dirac cone vertex (**b**), passing from an intermediate position (**c**). The color scale is normalized to the maximum intensity of the $\omega {<}0.4$ eV mode, with the red dots labeling the undamped plasmon resonances, and the dominant plasmon modes marked as in Fig. 5. The dashed lines delimit the regions where one-electron excitations, involving the $\pi$ and $\pi ^{*}$ bands, occur around the K point (SPE$\pi$), and one-electron excitations, involving the $\sigma$ and $\sigma ^{*}$ bands, occur around the $\Gamma$ point (SPE$\sigma$).

Additional insights come from the zoom on the 2DP+$\sigma$P and $\sigma ^{\prime }$P structures, shown in Fig. 6a, for ${q}{<}0.08\,\mathrm{\text{\AA} }^{-1}$ along ${{\Gamma }\mathrm{{K}}}$, where we see that the highest peak propagates in the region where Dirac cone SPEs at the K point of the 1$^\mathrm{st}$BZ are absent, being mostly determined by the massless 2DP. Such a condition is confirmed by the energy position of the same mode in FGe with similar Fermi level shift relative to $E_\mathrm{{C}}$. Complementary, the lowest peak occurs in the region where $\Gamma$-point excitations are absent, being entirely determined by the massive $\sigma ^{\prime }$P.

A way to isolate the $\sigma$P mode is to drive the Fermi level of $\mathrm{{QFGe{\,}on{\,}MoS}}_{2}$ at the Dirac cone vertex, which reduces the massless plasmon to the tiny structure recorded in intrinsic FGe (Fig. 5a) and $\mathrm{{QFGe{\,}on{\,}AlN}}$ (Fig. 5b). This particular extrinsic condition is shown in the loss function of Fig. 6b, where the highest peak must be ascribed to the intraband massive plasmon, because it lies ${\sim }0.2$ eV above the expected massless 2DP energy (see Supplementary Information, Sec. III and Sec.IV).

As the Fermi level is lowered down from the Dirac cone vertex, the 2DP mode comes into play and interferes with the $\sigma$P mode, which is shown in Fig. 6c, where an intermediate extrinsic condition is considered, between the intrinsic and half-filled Dirac cone cases.

**Figure 7:**
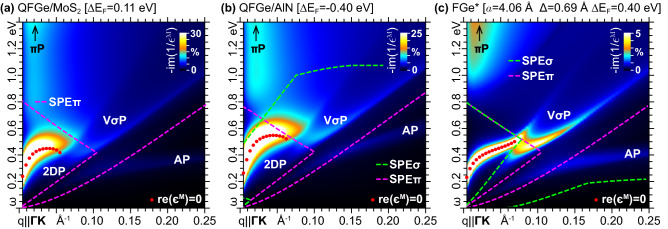
Loss function (in arb. units) of (**a**) $\mathrm{{QFGe{\,}on{\,}MoS}}_{2}$, (**b**) $\mathrm{{QFGe{\,}on{\,}AlN}}$, and (**c**) stretched FGe ($4.4\,\mathrm{\%}$ wider than the LDA geometry of Fig. 1a, defined by $a{=}4.06$ Å and $\Delta {=}0.69$ Å) for applied momentum transfers along ${{\Gamma }\mathrm{{K}}}$. The Fermi level is driven to the top of the $\sigma$-like bands in (**a**), (**b**), and to the bottom of the $\sigma ^{*}$-like band in (**c**). The color scale is normalized to the highest FIR-MIR peak, with the undamped plasmon resonances, the 2DP mode, and the SPE region delimiters marked as in in Fig. 6. AP denotes a weak acoustic modes of the Dirac cone charge-carriers activated by the specific direction of the incident momentum (${\mathbf{q}}{\parallel }{{\Gamma }\mathrm{{K}}}$). V$\sigma$P in (**c**) labels novel massive mode disjoint from the massless 2DP and AP.

Another important situation pops up when $E_\mathrm{{F}}$ is driven around $E_{\sigma }^{\Gamma }$, as displayed in Fig. 7a, where a different form of 2DP-$\sigma$ correlation emerges, with the $\sigma$P being shifted in momentum space and assuming a V-shaped feature for $q{\gtrsim }0.1$Å$^{-1}$. This peculiar massless-massive plasmon interaction can be controlled by finetuning of $E_\mathrm{{F}}$ and geometry driven band distortion. Indeed, a similar scenario appears in $\mathrm{{QFGe{\,}on{\,}AlN}}$, as detailed in Fig. 7b, where the different QFGe geometry offers a similar Fermi level positioning relative to the $\sigma$-like bands, with respect to $\mathrm{{QFGe{\,}on{\,}MoS}}_{2}$. Additionally, the $\sigma$P becomes competitive with the 2DP when the $\sigma ^{*}$-like band comes into play, as in the case of FGe stretched by $4.4\,\mathrm{\%}$ relative to its LDA geometry^68,69^, where the extrinsic condition ${{\Delta }E_\mathrm{{F}}}{=}0.4$ eV leads to the strongly correlated 2DP-V$\sigma$P feature given in Fig. 7c.

**Figure 8:**
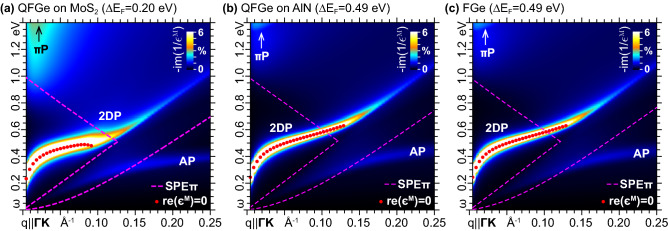
Loss function (in arb. units) of (**a**) $\mathrm{{QFGe{\,}on{\,}MoS}}_{2}$, (**b**) $\mathrm{{QFGe{\,}on{\,}AlN}}$, and (**c**) FGe for applied momentum transfers along ${{\Gamma }\mathrm{{K}}}$. The Fermi level is driven well above the top of the $\sigma$-like bands, at 0.49eV with respect to the Dirac cone vertex, in such a way that only the massless 2DP and AP modes are present. The color scale is normalized to the highest FIR-MIR peak, with the undamped plasmon resonances and the SPE$\pi$ region delimiters marked as in in Figs. 6–7.

Finally, when $E_\mathrm{{F}}$ is kept within the $\sigma$-$\sigma ^{*}$ band gap at $\Gamma$, the massive plasmon is excluded, which opens up the typical scenario of group IV 2D honeycomb lattices, where the 2DP propagates out-of-phase with square-root-like dispersion, in parallel with a smaller in-phase acoustic plasmon (AP) triggered by momentum transfers along selected directions, e.g., ${\mathbf{q}}{\parallel }{{\Gamma }\mathrm{{K}}}$^37,38^. The 2DP and AP modes are shown in Fig. 8, with the loss functions of the FGe and QFGe monolayers computed at the same Fermi level shift relative to the Dirac cone vertex. The massless plasmons are also exclusively implicated in the FIR-NIR dielectric response of FGe and QFGe, within a broad range of extrinsic conditions (see Supplementary Information, Sec. III and Sec.IV).

On the other hand, in both the stretched FGe and QFGe geometries, the AP mode can be detected along with the 2DP and massive modes for momentum transfers along ${{\Gamma }\mathrm{{K}}}$, see Fig. 7. The AP disappears for momentum transfers along ${{\Gamma }\mathrm{{M}}}$, as already observed in graphene and silicene^37,38^ (see also Supplementary Information, Sec. IV).

### Many-body quasiparticle and excitonic effects

To improve the reliability of the above presented analysis, we first estimated the role of GW quasiparticle corrections to the LDA band energies^45–50^. We focused in particular on FGe and $\mathrm{{QFGe{\,}on{\,}MoS}}_{2}$ as complementary examples of purely massless and massless-massive plasmonic substrates, under specific extrinsic regimes. Then, we applied the TDDFT-RPA machinery and calculated the dielectric properties of both overlayers by replacing the LDA band energies with the GW corrected band energies, while leaving unaltered the systems’ wave functions.

**Figure 9:**
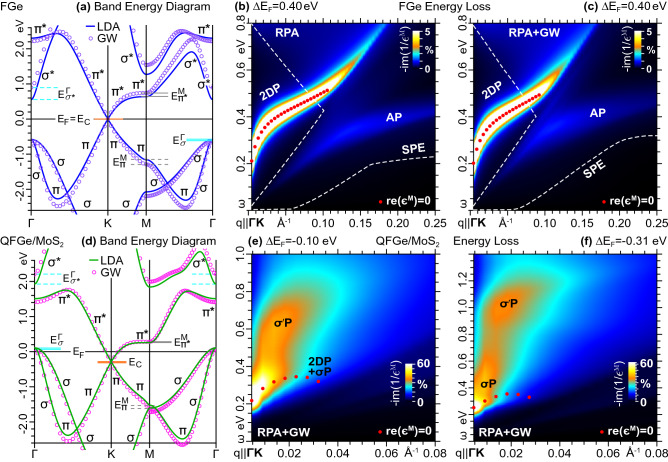
(**a**) LDA *vs* GW bands and (**b**), (**c**) loss function (in arb. units) of FGe for ${{\Delta }E_\mathrm{{F}}}{=}0.4\,\mathrm{eV}$, obtained (**b**) with the LDA electronic structure (within the TDDFT-RPA approach) and (**c**) with the GW correction to the LDA energies (within the TDDFT-RPA+GW approach). The dashed white lines delimit the full SPE region. (**d**) LDA *vs* GW bands and (**e**), (**f**) loss function (in arb. units) of $\mathrm{{QFGe{\,}on{\,}MoS}}_{2}$, obtained with the GW corrected energies for (**e**) ${\Delta }E_\mathrm{{F}}{=}{-}0.10$ eV and (**f**) ${-}0.31$ eV (within the TDDFT-RPA+GW approach). The density maps in Figs. 6a and 7a are the TDFT-RPA counterparts of (**e**) and (**f**). The color scale is normalized to the highest FIR-MIR peak, with the undamped plasmon resonances, dominant plasmon modes, and other energy labels marked as in Figs. 5–8.

The GW bands of FGe, shown in Fig. 9a, exhibit a significant narrowing of the Dirac cone relative to the LDA bands, equivalent to an increased slope of 0.30, in units of the Bohr velocity. A similar though lower increase rate has been reported for the Fermi velocity in graphene^48^. Additionally, the GW energy of the top $\sigma$-like states are downshifted by ${\sim }0.04\,\mathrm{eV}$ with respect to the LDA value. All other energies, related with the two highest occupied and lowest unoccupied bands, are reported to differ by a maximum value of 0.4eV.

Fig. 9b,c display the loss function of extrinsic FGe, respectively obtained within the RPA and the above outlined RPA+GW frameworks. The considered Fermi level shifting, ${\Delta }E_\mathrm{{F}}=0.4$ eV, activates highly resolved 2DP and AP modes, excluding the massive modes. Indeed, the latter would be present under extreme doping or gating conditions, being such that $-0.55{\le }{\Delta }E_\mathrm{{F}}{\le }0.58$ eV with the LDA energies, and $-0.60{\le }{\Delta }E_\mathrm{{F}}{\le }0.95$ eV including the GW quasiparticle corrections. By comparing Fig. 9b with Fig. 9c, we see that the RPA and RPA+GW loss spectra exhibit qualitatively similar propagation and damping trends, though the change in Fermi velocity results in smaller amounts of charge carriers and narrower SPE regions.

On the other hand, as reported in Fig. 9d, both the Dirac cone vertex and the highest $\sigma$-like states of $\mathrm{{QFGe{\,}on{\,}MoS}}_{2}$ experience very small changes, below 0.04eV. Additionally, the GW Dirac cone slope is increased by $6\,\mathrm{\%}$, while the GW mass of the $\sigma$-like charge carriers are practically identical to the corresponding LDA values. All other energies, within the two highest occupied and lowest unoccupied bands, differ by a maximum value of 0.3eV.

Nonetheless, the peculiar RPA plasmon structures of Figs. 6a and 7a correspond to the RPA+GW scenarios depicted in Figs. 9e,f, after ${{\Delta }E_\mathrm{{F}}}$ is adjusted to compensate the differences in the GW and LDA values of the Dirac cone energy and Fermi velocity. In particular, the extrinsic condition ${{\Delta }E_\mathrm{{F}}}=-0.10$ eV in Fig. 9e drives the intrinsic Fermi level at the same position, relative to the Dirac cone vertex, as Fig. 6a, yielding almost identical concentrations of massless and massive charge carriers. Furthermore, the extrinsic condition ${{\Delta }E_\mathrm{{F}}}=-0.32$eV in Fig. 9f drives the intrinsic Fermi level at the Dirac cone vertex, as in Fig. 7a. Thus, the expected tunability of the 2DP, AP, and $\sigma$P modes can be improved by correcting the LDA band energies of the germanene monolayers with more accurate predictions coming from many-body correlations or experimental data.

As a final scrutiny, we estimated the role of excitonic effects on the dielectric properties of FGe and $\mathrm{{QFGe{\,}on{\,}MoS}}_{2}$, using the BSE framework^52–54^, which we implemented with the bare, three-dimensional (3D) Coulomb potential. In this, we considered a minimum applied momentum of 2.5-$2.6{\times }10^{-3}\,\mathrm{\text{\AA} }^{-2}$, equivalent to a reduced accuracy on the 1$^\mathrm{st}$BZ sampling by one tenth, with respect to our RPA and RPA+GW calculations. To suppress the increased noise in the BSE spectra, we adopted a lifetime broadening parameter being five times larger than the RPA and and RPA+GW spectra shown above. We further performed control tests within the RPA approach, here denoted RPA$^{*}$, using the 3D Coulomb potential and the same resolutions as the BSE calculations.

**Figure 10:**
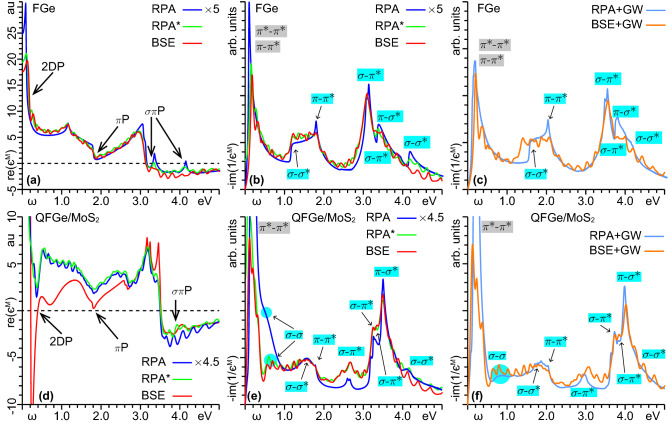
Macroscopic permittivity of (**a**)–(**c**) FGe and (**d**)–(**f**) $\mathrm{{QFGe{\,}on{\,}MoS}}_{2}$ in intrinsic conditions (${{\Delta }E_\mathrm{{F}}}{=}0$), as obtained within (**a**), (**b**), (**d**), (**e**) the RPA and BSE approximations, and (**c**), (**f**) the RPA+GW and BSE+GW approximations. RPA$^{*}$ denote the RPA calculations obtained with the bare Coulomb potential and the same resolution as the BSE computations. In (**a**), (**d**), the resonant energies of the 2DP, $\pi$P and $\sigma$P are spotted in changes of sign, or attempted change of sign of $\mathrm{re}(\epsilon ^{{ \textsc {m}}})$, with the linear vertical scale expressed in au. Unlike Fig. 3, the macroscopic imaginary permittivities in (**b**), (**c**), (**e**), (**f**) are given on a linear vertical scale, expressed in arb. units. The comparison of the RPA$^{*}$ and BSE results in (**b**), (**e**) suggests that the sub-peaks in (**b**), (**c**), (**e**), (**f**), superimposed to the main intraband and interband absorption peaks, already identified within the RPA or RPA+GW treatments, are an artifact of the reduced resolution in BSE computations. On the other hand, the RPA and RPA+GW computations presented here have a much more accurate resolution on the 1$^\mathrm{st}$BZ, thus giving a more reliable prediction of the FIR-NIR feature.

Fig. 10 reports the BSE and BSE+GW macroscopic permittivity of FGe and $\mathrm{{QFGe{\,}on{\,}MoS}}_{2}$ against their RPA, RPA$^{*}$, and RPA+GW counterparts. To ease the comparison, an overall renormalization factor is applied to the RPA and RPA+GW results, to compensate for the use of the 3D Coulomb interaction without our 2D truncation in the RPA$^{*}$, BSE and BSE+GW calculations.

Apart from the different broadening in the real and imaginary permittivity parts, we find that the plasmon resonances, of the 2DP, $\pi$P, and $\sigma \pi$P modes, and the intraband and interband peak positions are well defined in both approaches, being in close agreement with each other, within a maximum difference below 0.02 eV. We also notice that the overall VIS-NUV features of the RPA and BSE permittivities, or the RPA+GW and BSE+GW permittivities, have very similar trends.

At a closer look, the comparison of the BSE and the control RPA$^{*}$ calculations suggests that excitonic effects may play a non-negligible role at lower and higher ends of the optical band. More importantly, the MIR-NIR absorption peaks in the BSE and BSE+GW approximation suffer from the low resolution on the 1$^\mathrm{st}$BZ sampling.

Additionally, the intraband and interband absorption peaks, plus related plasmon modes of the metal states in $\mathrm{{QFGe{\,}on{\,}MoS}}_{2}$, are confirmed to exist with both the RPA and BSE approaches, with the interband $\sigma$-$\sigma ^{*}$ feature being correctly described with the RPA+GW and BSE+GW formalisms. We can thus safely conclude that the inclusion of excitonic effects confirms the existence of a competitive interplay of massive and massless plasmon in germenene sheets with selected geometries.

## Discussion

The key role of optical plasmons in light coupling has been long identified in a wide variety of nano-objects^70–81^ and fully elucidated in simple 2D honeycomb materials^35–42^.

In the present study, we have singled out the additional versatility of germanene monolayers and related interfaces^87^, whose multiple light-matter modes are strongly dependent on both geometry and charge carrier concentration. We remark that the latter is a novel feature, which marginally involve other 2DDMs, and as such it requires an improved analysis, in line with what has been reported here, for a correct tuning of the input parameters of possible germanene-based devices.

Our investigation has provided a complete picture of the massless and differently massive charge density waves in currently synthesized germanene sheets, outlining a unique playground of collective states in 2D quantum matter that can be manipulated for on-demand optoelectronic or plasmonic purposes.

This may serve to embed germanene-based building blocks in novel van der Waals heterostructures, or implement 2D platforms for extreme light confinement, compatible with standard semiconductor technology.

## Methods

The TDDFT and GW calculations were carried out using a package of Open-MP/MPI Fortran codes, developed by M.P. and A.S., which were interfaced with the DFT output from Abinit^57,58^, and implemented in one of the high-performance computing facilities provided by the CINECA consortium (Italy).

### Density functional calculations

As a routinely established framework, our TDDFT-RPA calculations^40–44^ required a preliminary DFT step to access the ground state of FGe, $\mathrm{{QFGe{\,}on{\,}AlN}}$, and $\mathrm{{QFGe{\,}on{\,}MoS}}_{2}$, which we reconstructed by the PW pseudopotential approach^57,58^. This involves the basis set of space functions $\mathrm{{PW}}_{\mathbf{k}+\mathbf{G}}={\Omega ^{-1/2}e^{\mathrm{{i}}({\mathbf{k}+\mathbf{G}})\cdot {\mathbf{r}}}}$, indexed by the wave vectors ${\mathbf{k}}$ of the 1$^\mathrm{st}$BZ and the reciprocal lattice vectors ${\mathbf{G}}$, in the normalization volume ${\Omega }$. Specifically, we expressed the outer electron properties of the three germanene phases, artificially replicated in three-dimensions, in terms of KS energy levels ${\varepsilon _{{\nu }{\mathbf{k}}}}$ associated to single-particle states ${|{\nu }{\mathbf{k}}\rangle }$, accordingly expanded in the $\mathrm{PW}_{\mathbf{k}+\mathbf{G}}$ basis, where $\nu$ denotes the band number. We adopted an LDA-scheme, based on the Teter-Pade exchange-correlation (xc) functional^59,60^ combined with a norm conserving Troullier-Martins pseudopotential^61^. In all cases, we chose a periodic out-of-plane separation *L* of 20 Å between the replicated germanene planes, which resulted in numerically negligible KS wave functions at distances larger than ${\sim }6$ Å from each replica (within an error of $10^{-6}$ %). As for the in-plane geometry, our PW-LDA optimizations provided the two defining parameters of the FGe lattice, namely, the hexagonal lattice constant $a{=}3.970$ Å and the buckling distance $\Delta {=}0.640$ Å, between the two germanium atoms of the crystal basis [Fig. 1], which turned out to be consistent with the literature^68,69^. Within the same PW-LDA scheme, we simulated the two QFGe geometries, using the experimentally derived values $a=3.928$ Å, $\Delta =0.705$ Å for AlN-distorted germanene^23^ and $a=3.820$ Å, $\Delta =0.860$ Å for $\mathrm{{MoS}}_{2}$-distorted germanene^24^, which respectively correspond to a $2.1\,\mathrm{\%}$ and $7.4\,\mathrm{\%}$ lattice compression, relative to FGe in the LDA geometry. In the self-consistent runs, first we limited the number of PWs in ${|{\nu }{\mathbf{k}}\rangle }$ to ${\sim }10^{4}$, by the energy cutoff $|{\mathbf{k}}+{\mathbf{G}}|^2/2<25\,\mathrm{Ha}$. Then, we sampled the 1$^\mathrm{st}$BZ (Fig. 1e) with a Monkhorst-Pack (MP) grid^82^ of $90{\times }90{\times }1$ $\mathbf{k}$ points. Next, we fixed an energy convergence criterion of $10^{-12}\,\mathrm{Ha}$. The resulting LDA bands and DOS are reported in Fig. 1a–c, while the charge carrier concentrations are given in Fig. 2.

For testing purposes, we compared the LDA results with those obtained from the generalized gradient approximation (GGA), based on the Perdew-Burke-Ernzerhof xc functional^83^ combined with a Vanderbilt norm-conserving pseudopotential^84^. Accordingly, we used the GGA optimized parameters $a{=}4.061$ Å and $\Delta {=}0.695$ Å for FGe^69^, while leaving unaltered the experimentally derived parameters of the QFGe structures^23,24^. We recorded minor differences between the LDA and GGA electronic structures, in an energy window of ${\sim }2.5$ eV around the Fermi level (see Supplementary Information, Sec. V), which covers electronic transitions and collective excitations in the FIR to VIS range, being the core interest of our study.

### Time-dependent density functional approach

As a second step of the TDDFT machinery, we refined the KS eigensystem $\{{\varepsilon _{{\nu }{\mathbf{k}}}},{|{\nu }{\mathbf{k}}\rangle }\}$ in non-self-consistent runs on an MP grid of $720{\times }720{\times }1$ **k** points. We further included more than 10 unoccupied bands to encompass all possible SPEs below ${\sim }5\,\mathrm{eV}$. Then, we plugged this information into the Adler-Wiser formula^31,32^

$$\chi _{{\mathbf{G}}{\mathbf{G}^{\prime }}}^{0}= \frac{2}{\Omega } \sum _{{\mathbf{k}},\nu ,{\nu ^{\prime }}} \frac{(f_{{\nu }{\mathbf{k}}}-f_{{\nu ^{\prime }}{{\mathbf{k}}+{\mathbf{q}}}})\rho _{{\nu }{\nu ^{\prime }}}^{{\mathbf{k}}{\mathbf{q}}}({\mathbf{G}})\rho _{\nu {\nu ^{\prime }}}^{{\mathbf{k}}{\mathbf{q}}}({\mathbf{G}^{\prime }})^{*}}{\omega +\varepsilon _{{\nu }{\mathbf{k}}}-\varepsilon _{{\nu ^{\prime }}{{\mathbf{k}}+{\mathbf{q}}}}+\mathrm{{i}}\eta },$$

to obtain the *non-interacting* density-density response function of the KS electrons. Here, ${\chi _{{\mathbf{G}}{\mathbf{G}^{\prime }}}^{0}}$ is triggered by an optical photon of in-plane momentum ${\mathbf{q}}$ and energy $\omega$. $f_{{\nu }{\mathbf{k}}}$ and $f_{{\nu ^{\prime }}{{\mathbf{k}}+{\mathbf{q}}}}$ respectively denote the Fermi-Dirac occupations of the energy levels $\varepsilon _{{\nu }{\mathbf{k}}}$ and $\varepsilon _{{\nu ^{\prime }}{{\mathbf{k}}+{\mathbf{q}}}}$, with the factor of 2 arising from electron spin degeneracy. $\rho _{{\nu }{\nu ^{\prime }}}^{{\mathbf{k}}{\mathbf{q}}}({\mathbf{G}}){=}\langle {\nu }{\mathbf{k}}|e^{{-}\mathrm{{i}}({\mathbf{q}}+{\mathbf{G}})\cdot \hat{\mathbf{r}}} |{\nu ^{\prime }}{{\mathbf{k}}+{\mathbf{q}}}\rangle$ and $\rho _{\nu {\nu ^{\prime }}}^{{\mathbf{k}}{\mathbf{q}}}({\mathbf{G}^{\prime }})^{*}$ are oscillator (or screened) matrix elements. Retarded propagation or damping is governed by the positive infinitesimal broadening $\eta$, replaced by $\eta =0.01\,\mathrm{eV}$ for numerical convenience.

Subsequently, we calculated the *interacting* density-density response function by the fundamental equation of TDDFT, namely, $\chi _{{\mathbf{G}}{\mathbf{G}^{\prime }}}{=}\chi _{{\mathbf{G}}{\mathbf{G}^{\prime }}}^{0}{+}[\chi ^{0}(v+f_{{ \textsc{xc}}})\chi ]_{{\mathbf{G}}{\mathbf{G}^{\prime }}}$. In the procedure, we neglected the exchange-correlation part $f_{{ \textsc {xc}}}$ of the interaction kernel $v+f_{{ \textsc {xc}}}$. Additionally, we replaced the *v*-operator with the RPA local kernel

$$v_{{\mathbf{G}}{\mathbf{G}^{\prime }}} = v_{{\mathbf{g}}{\mathbf{g}^{\prime}}}^{{2{\text{d}}}} \int_{{-L/2}}^{{L/2}} {dz} \int_{{ - L/2}}^{{L/2}} {dz^{\prime } } e^{{{\text{i}}(G_{z} z - G_{z}^{\prime } z^{\prime } ) - |{\mathbf{q}} + {\mathbf{g}}||z + z^{\prime } |}} ,$$

based on the 2D Coulomb potential $v_{{{\mathbf{gg}}^{\prime } }} ^{{2{\text{d}}}} = 2\pi \delta _{{{\mathbf{gg}}^{\prime } }} /|{\mathbf{q}} + {\mathbf{g}}|$, with ${\mathbf{g}}{,}{\;}{\mathbf{g}^{\prime }}$ and ${G_{z}}{,}{\;}{G_{z}^{\prime }}$ respectively labeling the in-plane and out-of-plane components of $\mathbf{G}$ and $\mathbf{G}^{\prime }$. The advantage of $v_{\mathbf{G}\mathbf{G}^{\prime }}$ is the deletion of redundant density-density interactions among the replicated monolayer slabs^35–44^, which provides far more accuracy to the low-momentum dielectric response of 2D materials with respect to the usual Coulomb potential ${v_{{\mathbf{G}}{\mathbf{G}^{\prime }}}^{{ \textsc {3d}}}}=\lim \limits _{L\rightarrow \infty }v_{\mathbf{G}\mathbf{G}^{\prime }}{=}{4\pi \delta _{{\mathbf{G}}{\mathbf{G}^{\prime }}}}/{|{\mathbf{q}}+{\mathbf{G}}|^{2}}$.

Next, we treated the fundamental equation of TDDFT at the RPA level in the small interaction limit, to obtain $\chi _{{\mathbf{G}}{\mathbf{G}^{\prime }}}{=}[\chi ^{0}(1{+}v\chi ^{0})^{{-}1}]_{{\mathbf{G}}{\mathbf{G}^{\prime }}}$^33,34^. Finally, we determined the inverse dielectric matrix $\epsilon _{{\mathbf{G}}{\mathbf{G}^{\prime }}}^{-1}{=}\delta _{{\mathbf{G}}{\mathbf{G}^{\prime }}}{+}(v\chi )_{{\mathbf{G}}{\mathbf{G}^{\prime }}}$, which gives access to the macroscopic permittivity $\epsilon ^{{ \textsc {m}}}{=}1/\epsilon _{\mathbf{0 0}}^{{-}1}$. In doing so, we efficiently included crystal-local field effects, associated with the off-diagonal elements of $\chi _{{\mathbf{G}}{\mathbf{G}^{\prime }}}^{0}{,}{\;}\chi _{{\mathbf{G}}{\mathbf{G}^{\prime }}}{,}{\;}\mathrm{and}{\;}\epsilon _{{\mathbf{G}}{\mathbf{G}^{\prime }}}^{-1}$^85^, by restricting the calculation to the smallest ${\sim }100$ ${\mathbf{G}}$-vectors of the form $(0,0,G_{z})$.

The TDDFT-RPA framework just outlined can be tuned by adjusting the occupation factors in $\chi _{{\mathbf{G}}{\mathbf{G}^{\prime }}}^{0}$ to account for changes in both Fermi level and temperature. Working at room temperature, we simulated the intrinsic and extrinsic dielectric responses of the germanene sheets by replacing ${E_\mathrm{{F}}}$ with ${E_\mathrm{{F}}}{+}{{\Delta }E_\mathrm{{F}}}$ in $f_{{\nu }{\mathbf{k}}}$ and $f_{{\nu ^{\prime }}{{\mathbf{k}}+{\mathbf{q}}}}$, while leaving unaltered the KS energies, $\varepsilon _{{\nu }{\mathbf{k}}}$ and $\varepsilon _{{\nu ^{\prime }}{{\mathbf{k}}+{\mathbf{q}}}}$, and oscillator matrix elements, $\rho _{{\nu }{\nu ^{\prime }}}^{{\mathbf{k}}{\mathbf{q}}}({\mathbf{G}})$ and $\rho _{\nu {\nu ^{\prime }}}^{{\mathbf{k}}{\mathbf{q}}}({\mathbf{G}^{\prime }})^{*}$.

The main outputs from such calculations are given in Figs. 3–8. In particular, the optical absorption features were computed at the smallest sampled momenta allowed by the chosen MP-grid, namely, $q_{{\Gamma }\mathrm{{M}}}^0{=}\pi /(180 \sqrt{3} a)$, along ${{\Gamma }\mathrm{{M}}}$, and $q_{{\Gamma }\mathrm{{K}}}^0{=}\pi /(180 a)$, along ${{\Gamma }\mathrm{{K}}}$, with *a* the lattice constant. The ${\mathbf{q}}{\parallel }{{\Gamma }\mathrm{{M}}}$ absorption spectra of Fig. 3 were found to be identical to the ${\mathbf{q}}{\parallel }{{\Gamma }\mathrm{{K}}}$ absorption spectra, within the numerical errors. The energy loss spectra of Figs. 4–8 were computed over a broad range of sampled momenta $q{=}q_{{\Gamma }\mathrm{{M}}}^0$-$100\,q_{{\Gamma }\mathrm{{M}}}^0$, along ${{\Gamma }\mathrm{{M}}}$, and $q{=}q_{{\Gamma }\mathrm{{K}}}^0$-$60\,q_{{\Gamma }\mathrm{{K}}}^0$, along ${{\Gamma }\mathrm{{K}}}$.

### GW calculations

We probed the accuracy of our TDDFT-LDA-RPA framework by implementing the simplest first-order GW expansion of the self-energy $\Sigma _{\nu \mathbf {k}}$, around the LDA xc potential $v_{\nu \mathbf {k}}^{{ \textsc {lda}}}$^49^. Specifically, we computed the quasiparticle energy corrections

$$\varepsilon _{\nu \mathbf {k}}^{{ \textsc {gw}}}=\varepsilon _{\nu \mathbf {k}} +\left. \frac{\Sigma _{\nu \mathbf {k}}(\omega )-v_{\nu \mathbf {k}}^{{ \textsc {lda}}}}{1-\partial \Sigma _{\nu \mathbf {k}}(\omega )/\partial \omega } \right| _{\omega =\varepsilon _{\nu \mathbf {k}}},$$

to the unperturbed LDA energies $\{\varepsilon _{\nu \mathbf {k}}\}$ of FGe and QFGe. Accordingly, we evaluated the static, exchange part of the total self-energy

$$\Sigma _{\nu \mathbf {k}}^{{ \textsc {x}}}=-\frac{1}{\Omega } \sum \limits _{\nu ^{\prime }}^{{ \textsc {occ}}}\sum _{\mathbf {q}} \sum _{\mathbf {G,G}^{\prime }}\rho _{\nu \nu ^{\prime }}^{\mathbf {k}\mathbf {q}}(\mathbf {G})\, \rho _{\nu \nu ^{\prime }}^{\mathbf {k}\mathbf {q}}(\mathbf {G}^{\prime })^{*}\, v_{\mathbf {G}\mathbf {G}^{\prime }}(\mathbf {q}),$$

with cutoffs of 5Ha on the unperturbed KS wave functions and 10Ha on the $\mathbf {G}$ vectors. As for the dynamic, correlated self-energy

$$\Sigma _{\nu \mathbf {k}}^{{ \textsc {c}}}(\omega )=\frac{i}{2\pi \Omega }\sum \limits _{\nu ^{\prime }}\sum _{\mathbf {q}}\sum _{\mathbf {G,G}^{\prime }}\rho _{\nu \nu ^{\prime }}^{\mathbf {k}\mathbf {q}}(\mathbf {G})\,\rho _{\nu \nu ^{\prime }}^{\mathbf {k}\mathbf {q}}(\mathbf {G}^{\prime })^{*}\, v_{\mathbf {G}\mathbf {G}^{\prime }}(\mathbf {q})\,J_{\mathbf {G}\mathbf {G }^{\prime }}^{\nu ^{\prime }\mathbf {k-q}}(\mathbf {q},\omega )$$

we reduced the $\mathbf {G}$-vector cutoff to 5Ha, and replaced the *J* kernel with a plasmon pole model^47^. We found well converged results using a 72${\times }$72${\times }$1 MP grid to represent the unperturbed PW-LDA eigensystem $\{\varepsilon _{\nu \mathbf {k}},|\nu \mathbf {k}\rangle \}$ , over a total of 50 occupied and unoccupied bands. We further adopted the 2D-truncated local kernel of our TDDFT-RPA approach for the $v_{\mathbf {G}\mathbf {G}^{\prime }}$ matrix elements. The GW bands from FGe and $\mathrm{{QFGe{\,}on{\,}MoS}}_{2}$ are respectively reported in Fig. 9a,d, in comparison with the LDA bands of Fig. 1a,c.

As a final exploration, we implemented a modified TDDFT machinery, by replacing the LDA energies, sampled over the 720$\times$720$\times$1 MP-grid, with the GW energies of Fig. 9a,d, interpolated over the 720$\times$720$\times$1 MP-grid. In this way, we obtained the RPA+GW loss spectra of Fig. 9c–f.

### BSE calculations

We further compared our RPA and RPA+GW permittivity calculations with the macroscopic dielectric function

$$\epsilon _{{{ \textsc {bse}}}}^{M}=1-v_{\mathbf {00}}^{{{ \textsc {3d}}} }\sum \limits _{\nu \bar{\nu }\mathbf {k}}\sum \limits _{\nu ^{\prime }\bar{\nu } ^{\prime }\mathbf {k}^{\prime }}\rho _{{\nu }\bar{\nu }}^{{\mathbf {k}}{\mathbf { q}}}({\mathbf {0}})\rho _{\bar{\nu }^{\prime }{\nu ^{\prime }}}^{{\mathbf {k}} ^{\prime }{\mathbf {q}}}({\mathbf {0}})^{*}\sum _{\lambda }\frac{ a_{{\nu }\bar{\nu }{\mathbf {k}}}^{\lambda }a_{\bar{\nu }^{\prime }{\nu ^{\prime }\mathbf {k}}^{\prime }}^{\lambda *}}{\omega -\varepsilon _{\lambda }+ i\eta },$$

obtained from the BSE and BSE+GW approximations, as implemented in the YAMBO code^86^, which relies on the bare 3D Coulomb potential elements $v_{\mathbf {00}}^{{ \textsc {3d}}}=4\pi /|{\mathbf{q}}|^2$. Accordingly, we refined the DFT-LDA electronic structures of the monolayers on an MP grid of 72${\times }$72${\times }$1, thus adopting a reduced resolution on the transferred momenta by one tenth, relative to our TDDFT-RPA computations. To compensate for the lack in resolution, and reduce the noise in the behavior of $\epsilon _{{{ \textsc {bse}}}}^{M}$ *vs* $\omega$, we adopted a broadening parameter $\eta$ of 0.05eV, that is five times larger than our TDFT+RPA computations. We then considered the lowest sampled momentum $q_{{\Gamma }\mathrm{{M}}}^1$ along ${{\Gamma }\mathrm{{M}}}$, in the above mentioned coarse grid, with $q_{{\Gamma }\mathrm{{M}}}^1{=}\pi /(18 \sqrt{3} a)$. Such a value is ten times larger than the optical momentum $q_{{\Gamma }\mathrm{{M}}}^0$ used to derive the absorption spectra in our TDDFT-RPA approach. Next, we computed the eigenvalues $\varepsilon _{\lambda }$ and eigenvectors $a_{{\nu }\bar{\nu }{\mathbf {k}}}^{\lambda }$ of the two-particle hamiltonian

$$h_{\nu \bar{\nu }\mathbf {k}}^{\nu ^{\prime }\bar{\nu }^{\prime }\mathbf {k} ^{\prime }}=(\varepsilon _{\nu \mathbf {k}}-\varepsilon _{\nu ^{\prime } \mathbf {k}})\delta _{\nu \bar{\nu }}\delta _{\nu ^{\prime }\bar{\nu }^{\prime }}\delta _{\mathbf {kk}^{\prime }}+(f_{\nu \mathbf {k}}-f_{\nu ^{\prime } \mathbf {k}})(2u_{\nu \bar{\nu }\mathbf {k}}^{\nu ^{\prime }\bar{\nu }^{\prime } \mathbf {k}^{\prime }}-w_{\nu \bar{\nu }\mathbf {k}}^{\nu ^{\prime }\bar{\nu } ^{\prime }\mathbf {k}^{\prime }}),$$

on a limited number of states, allowing the $\nu$, $\bar{\nu }$, $\nu ^{\prime }$, and $\bar{\nu }^{\prime }$ indexes to run over the three highest occupied and lowest unoccupied bands. Finally, we computed the electron-hole exchange interaction matrix elements

$$u_{\nu \bar{\nu }\mathbf {k}}^{\nu ^{\prime }\bar{\nu }^{\prime }\mathbf {k} ^{\prime }}=\frac{1}{\Omega }\sum \limits _{\mathbf {G\ne 0}}v_{\mathbf {GG}}^{{ { \textsc {3d}}}}\rho _{{\nu }\bar{\nu }}^{{\mathbf {k0}}}({\mathbf {0}})\rho _{\bar{ \nu }^{\prime }{\nu ^{\prime }}}^{{\mathbf {k}}^{\prime }{\mathbf {0}}}({ \mathbf {0}})^{*}$$

with ${\sim }10^4$ reciprocal lattice vectors, and the electron-hole attraction matrix elements

$$w_{\nu \bar{\nu }\mathbf {k}}^{\nu ^{\prime }\bar{\nu }^{\prime }\mathbf {k} ^{\prime }}=\frac{1}{\Omega }\sum \limits _{\mathbf {G,G}^{\prime }}v_{\mathbf { GG}^{\prime }}^{{{ \textsc {3d}}}}\epsilon _{\mathbf {GG}^{\prime }}^{-1}\rho _{{ \bar{\nu }}{\bar{\nu }^{\prime }}}^{{\mathbf {k}}{\mathbf {q}}}({\mathbf {G}} )^{*}\rho _{{\nu }{\nu ^{\prime }}}^{{\mathbf {k}}{\mathbf {q}}}({\mathbf {G }})\delta _{{\mathbf {kk+q}}}$$

with ${\sim }10^2$ reciprocal lattice vectors, to obtain the BSE permittivity of Fig. 10a,b,d,e. We further corrected the LDA band energies with suitable renormalization factors on the first conduction and first valence bandwidths, derived from our GW calculations. In this way we obtained the BSE+GW permittivity of Fig. 10c,f.

## Supplementary Information

Supplementary Information.

## Data Availability

The authors declare that the data supporting the findings of this study are available within the paper (and its Supplementary Information files). Further data, concerning the outputs and codes from DFT and TDDFT calculations, are also available from the corresponding author upon reasonable request.
