# Supplementary material for: Massive and massless plasmons in germanene nanosheets
Source: Sci Rep. 2022 Nov 3;12:18624. doi: 10.1038/s41598-022-23058-3 (PMC9633710; doi:10.1038/s41598-022-23058-3)
Supplement: Supplementary file 1 — Supplementary Information. [file 41598_2022_23058_MOESM1_ESM.pdf]

Michele Pisarra,[1] Cristian Vacacela Gomez,[2] and Antonello Sindona[3,1,*]
[1]*INFN, sezione LNF, Gruppo collegato di Cosenza, Cubo 31C, I-87036 Rende (CS), Italy*
[2]*Fac. de Cienc., Esc. Super. Politéc. de Chimborazo (ESPOCH), Riobamba EC-060155, Ecuador*
[3]*Dip. di Fisica, Università della Calabria, Via P. Bucci, Cubo 30C, I-87036 Rende (CS), Italy*
(Dated: October 31, 2022)

Supplemental Information

CONTENTS

SI. Projected Bands and DOS 1

SII. Joint DOS and Leading Vertical Transitions 3

SIII. Dielectric Properties of FGe 5

SIV. Dielectric Properties of QFGe on AlN and MoS_2 7

SV. PW-DFT calculations within the LDA and GGA 9

SI. PROJECTED BANDS AND DOS

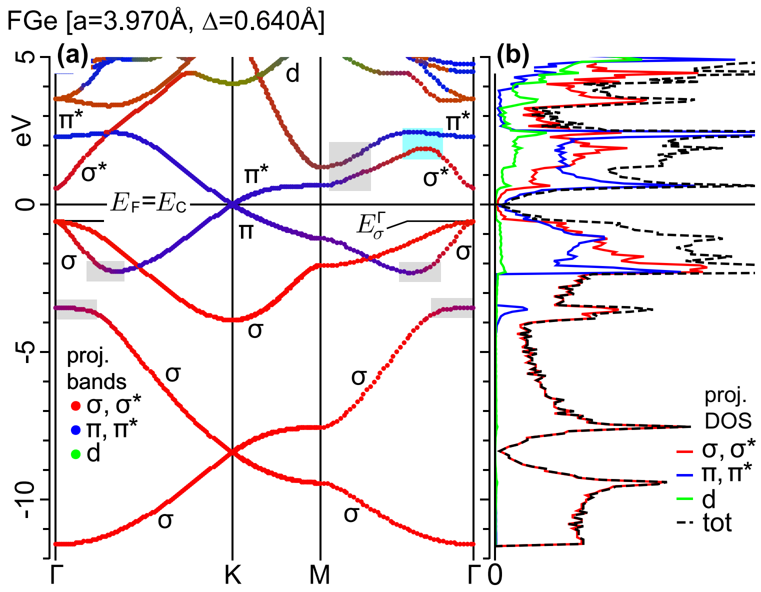

Figure S1
(a) LDA Band dispersions along the ΓKMΓ border of the irreducible 1^{st}BZ of FGe, decomposed according to their σ (s and in-plane p atomic orbitals), π (out-of-plane p atomic orbitals) and d characters. (b) DOS components of FGe, related to the projected bands in (a), and total DOS resulting from the full band dispersions of FGe, reported in Fig. 1(a) of the main text. The highest valence and lowest conduction bands have dominant π and π^* characters, respectively, over a wide region around the Dirac cone (with vertex $E_C = E_F$ at K). This region extends up to the flat parts of the two bands (at M), where the π-DOS and π^*-DOS display sharp peaks, associated to the VHSs. Within the same region, the second-highest valence and second-lowest conduction bands have dominant σ and σ^* characters, respectively. Unlike graphene and like silicene, the in-plane mirror symmetry is broken by structural buckling, which allows for sp^2 (σ, σ^*) to sp^3 (σ-π, σ^*-π^*) hybridization changes within the same band (gray boxes). As a result, the π-like band becomes σ-like around Γ, sharing the same character with the second-highest valence band. Additionally,

* antonello.sindona@fis.unical.it

the two highest valence bands tend to the degenerate value E_σ^Γ at Γ. Complementary, the π^* and σ^* characters of the lowest two conduction bands respectively switch to σ^* and π^*, around Γ, with an avoided crossing point along ΓM (cyan box). Finally, a d-like character is recorded in the unoccupied bands at energies larger than ∼3 eV above E_F. Nonetheless, the d-DOS has a small non-negligible effect on the occupied bands, starting from ∼-2 eV below E_F.

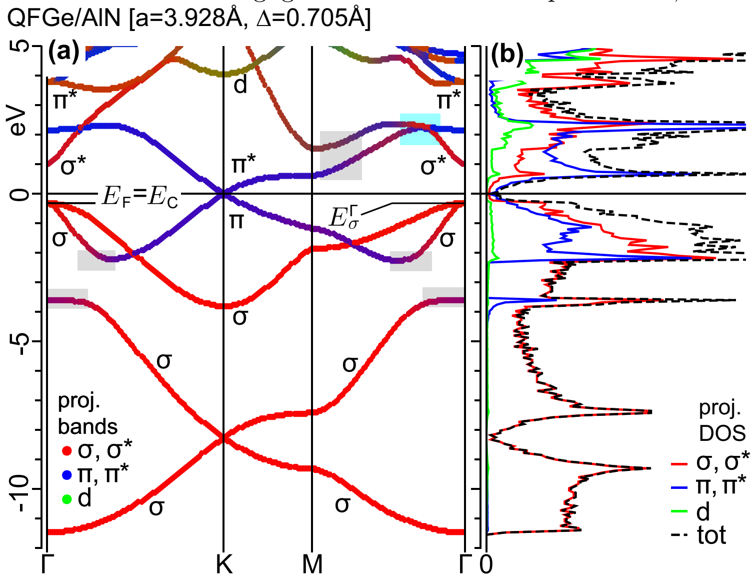

Figure S2

(a) LDA Band dispersions along ΓKMΓ and (b) related DOS of QFGe on AlN, decomposed as in Fig. S1, with same labels and shadings. All the bands and DOS components shown have similar behavior and identical character as FGe with $E_\mathrm{F} = E_\mathrm{C}$. Nonetheless, the two highest valence and the two lowest conduction bands are shifted up in energy by 0.3-0.4 eV around Γ. The 'avoided' crossing point of the σ^*-like and π^*-like bands of FGe along ΓM (Fig. S1(a)) becomes a crossing point in this QFGe monolayer (cyan box).

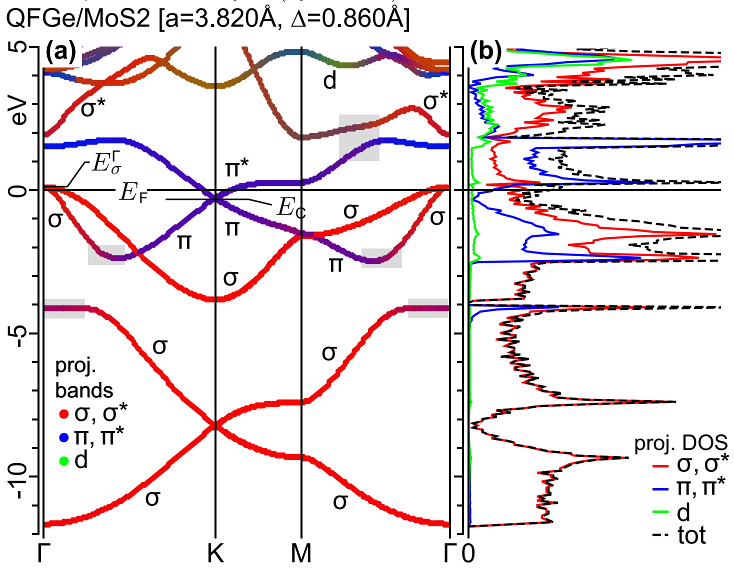

Figure S3

(a) LDA Band dispersions along ΓKMΓ and (b) related DOS of QFGe on MoS_2, decomposed as in Figs. S1 and S2, with same labels and shadings.

We notice an overall similarity of the bands and DOS components with FGe (Fig. S1) and QFGe on AlN (Fig. S2). Nonetheless, the two highest valence bands of this QFGe structure are further shifted up in energy, in such a way that the σ-like bands cross the Fermi level close to the Γ point, offering a bunch of unoccupied states just above E_F. As a result, this QFGe structure is a metal, with E_C shifted below E_F. In addition, the σ^*-like band lies above the π^*-like band.

SII. JOINT DOS AND LEADING VERTICAL TRANSITIONS

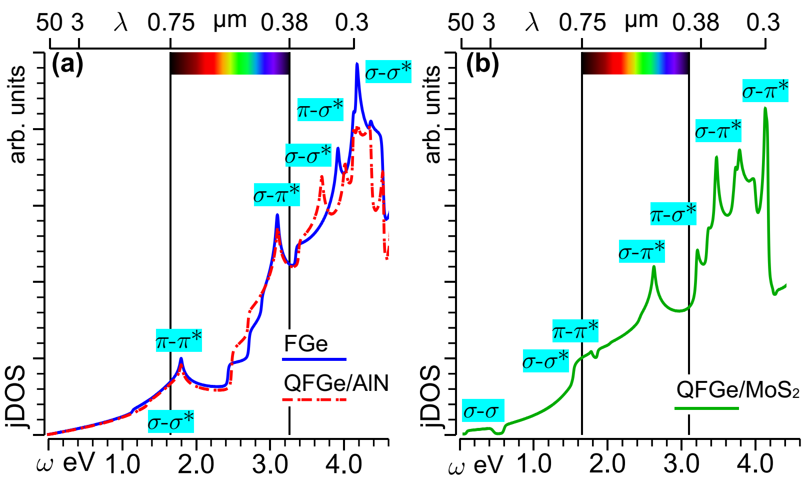

Figure S4
Joint density of states

$$\text{jDOS} = \frac{2}{\Omega}\sum_{\mathbf{k},\nu,\nu'} \text{im}\left(\frac{f_{\nu\mathbf{k}} - f_{\nu'\mathbf{k+q}}}{\omega + \varepsilon_{\nu\mathbf{k}} - \varepsilon_{\nu'\mathbf{k+q}} + \text{i}\eta}\right),$$

of (a) FGe, QFGe on AlN, and (b) QFGe on MoS_2, computed with the same input parameters as the absorption spectrum of Fig. 3 in the main text. In particular, the minimum probing momentum allowed by the $720\times720\times1$ MP-grid was applied, as detailed in the section ”Time-dependent density functional approach” of the main text. The jDOS provides complementary information on the leading interband SPEs that contribute to the absorption peaks at L-IR to M-UV wavelengths. As shown in Figs. S5-S7 below, these excitations involve the highest or second highest occupied σ- and π-like states and the lowest and second lowest unoccupied σ-, σ^*-, and π^*-like states. In particular, the L-IR to S-IR feature in QFGe on MoS_2 is due to SPEs between the two σ-like bands at the crossing point with the Fermi level of the metal, and corresponds the shoulder at 0.45 eV in the absorption lineshape of Fig. 3(b).

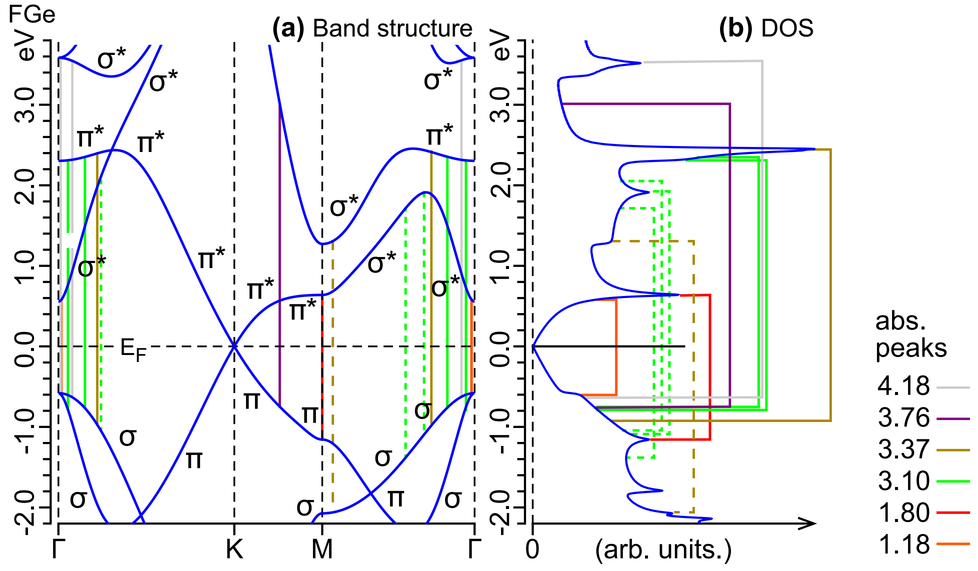

Figure S5
(a) Energy bands, along the high-symmetry ΓKMΓ contour of the IBZ, (b) density of states, and (a), (b) dominant vertical transitions contributing to the NIR-NUV peak structures in the absorption and jDOS spectra of FGe (see Figs. 1 and 3 of the main text, plus Fig. S4 above). These processes, which connect initial and final energy levels with high DOS, include: σ-σ^* SPEs around Γ, at 1.18 eV (NIR); π-π^* SPEs around M, at 1.80 eV (VIS); σ-π^* SPEs close to Γ, and, to a minor extent, σ-σ^* SPEs around the mid points of the ΓK and MΓ segments, at 3.10 eV (VIS-NUV); σ-π^* SPEs, around the mid points of the ΓK and MΓ segments, and σ-σ^* SPEs around M, at 3.37 eV (NUV); π-σ^* SPEs around the mid point of the KM segment, at 3.76 eV (NUV). σ-σ^* SPEs along the ΓK and MΓ segments, around

Γ at 4.18 eV (MUV).

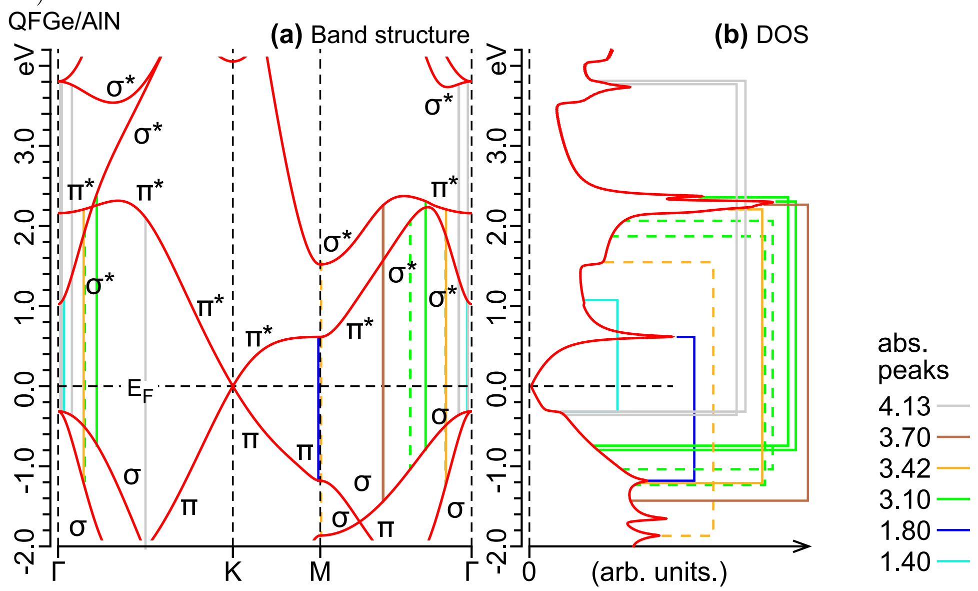

Figure S6

(a) Energy bands, along the high-symmetry ΓKMΓ contour of the IBZ, (b) density of states, and (a), (b) dominant vertical transitions contributing to the NIR-NUV peak structures in the absorption and jDOS spectra of QFGe on AlN, which include: σ-σ^* SPEs around Γ, at 1.40 eV (NIR); π-π^* SPEs around M, at 1.80 eV (VIS); σ-π^* SPEs, and, to a minor extent, σ-σ^* SPEs along the along the ΓK and MΓ lines, towards Γ at 3.10 eV (VIS-NUV). σ-π^* SPEs, along the ΓK and MΓ segments towards Γ, and σ-σ^* SPEs around M, at 3.42 eV (NUV); σ-σ^* SPEs along the ΓK and MΓ segments, around Γ at 4.13 eV (MUV).

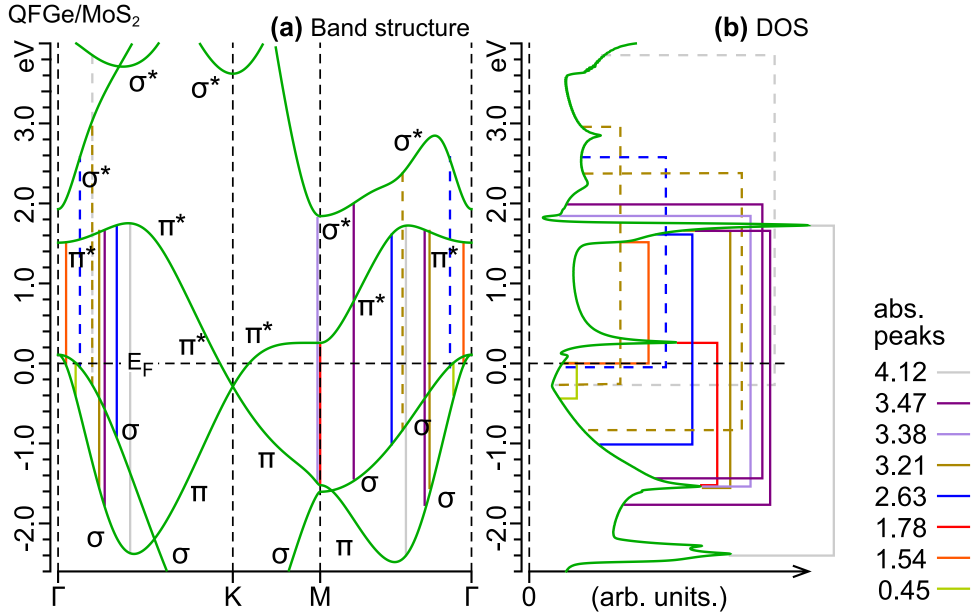

Figure S7

(a) Energy bands, along the high-symmetry ΓKMΓ contour of the IBZ, (b) density of states, and (a), (b) dominant vertical transitions contributing to the NIR-NUV peak structures in the absorption and jDOS spectra of QFGe on MoS_2, which include: σ-σ SPEs around Γ, at 0.45 eV (MIR-NIR); σ-σ^* SPEs around Γ, at 1.54 eV (NIR); π-π^* SPEs around M, at 1.80 eV (VIS); σ-π^* SPEs, and, to a minor extent, σ-σ^* SPEs along the ΓK and MΓ lines, around their mid points and Γ at 2.63 eV (VIS); σ-π^* SPEs around the mid points of the ΓK and MΓ lines, at 3.21 eV (NUV-VIS); π-σ^* SPEs around M, at 3.38 eV (NUV); σ-π^* SPEs along the ΓK and MΓ segments, towards Γ, and σ-σ^* SPEs along the MΓ segment, towards M, at 3.47 eV (NUV); π-σ^* SPEs around the mid points of the ΓK and MΓ lines, and σ-σ^* SPEs, along ΓK, closer to Γ, at 4.12 eV.

SIII. DIELECTRIC PROPERTIES OF FGE

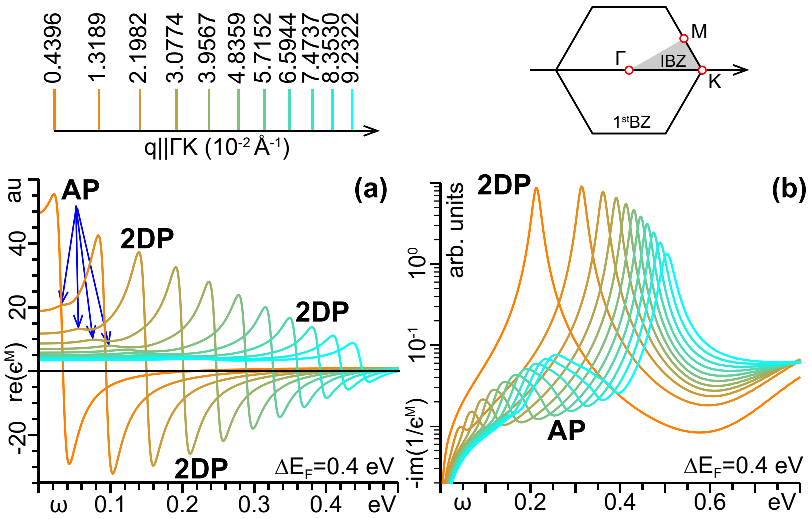

Figure S8
(a) Real macroscopic permittivity re(ϵ^{M}) (in au) and (b) loss function $\propto$-im($1/\epsilon^{\mathrm{M}}$) (in arb. units) of extrinsic FGe for small momentum transfers q<0.1 Å^{-1} along ΓK and a positive Fermi energy ΔE_{F} shift of 0.4 eV. In (a), the pairs of zeros of re(ϵ^{M}) at fixed **q** are associated to the undamped 2DP. The small features, visible for q<0.05 Å^{-1} (blue arrows), are hallmarks of the damped AP. In (b), the propagation of the 2DP is characterized by the main peaks of -im($1/\epsilon^{\mathrm{M}}$) that precede the highest zeros of re(ϵ^{M}). The secondary peaks of -im($1/\epsilon^{\mathrm{M}}$) define the AP features.

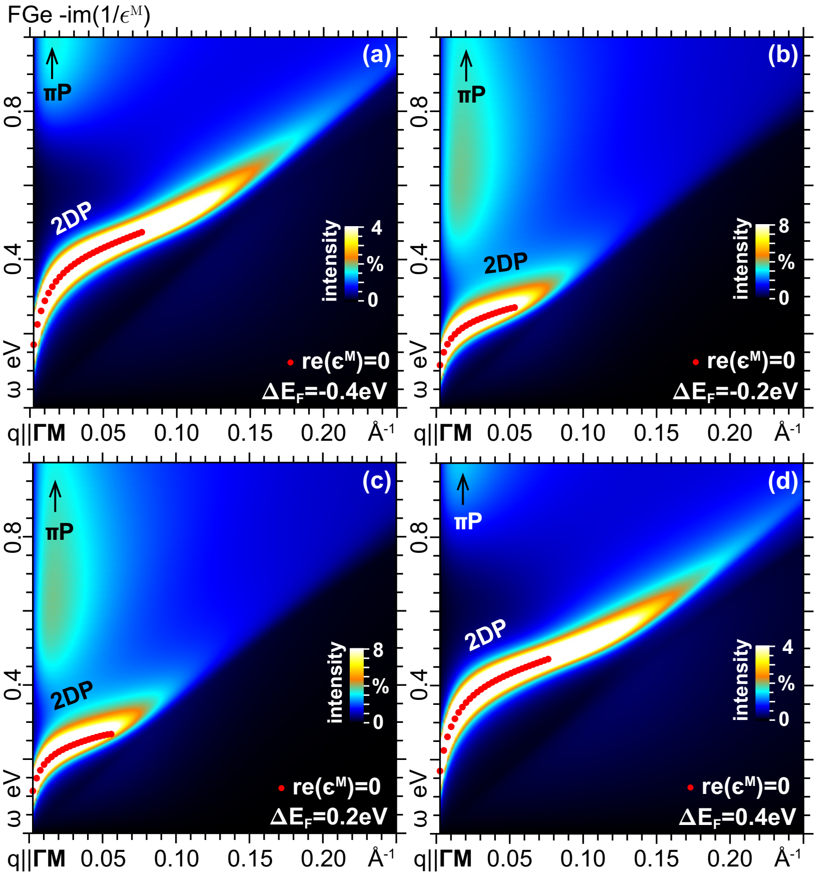

Figure S9
Loss function of FGe under different extrinsic (doping or gating) conditions, for momentum transfers **q** along ΓM. The intrinsic Fermi energy shifts $|\Delta E_{\mathrm{F}}|\leq$0.40 eV considered here are capable of activating a single sheet plasmon (2DP) with square-root-like dispersion over the MIR-NIR band. Indeed, this oscillation of the massless charge carriers can be individually tuned by changing the Fermi level position in the range where the σ-like or σ^*-like states are left inert,

namely $-0.55 \leq \Delta E_{\mathrm{F}} \leq 0.58\,\mathrm{eV}$, as also suggested by the associated band dispersions (see Fig. 1a of the main text). Graphene and silicene have similar responses for $\mathbf{q}||\Gamma\mathrm{M}$ and equivalent extrinsic conditions[37,38]. The same settings as Figs. 5-8 of the main text are used, with the color scale normalized to the highest FIR-MIR peak, the red dots denoting the plasmon resonance points.

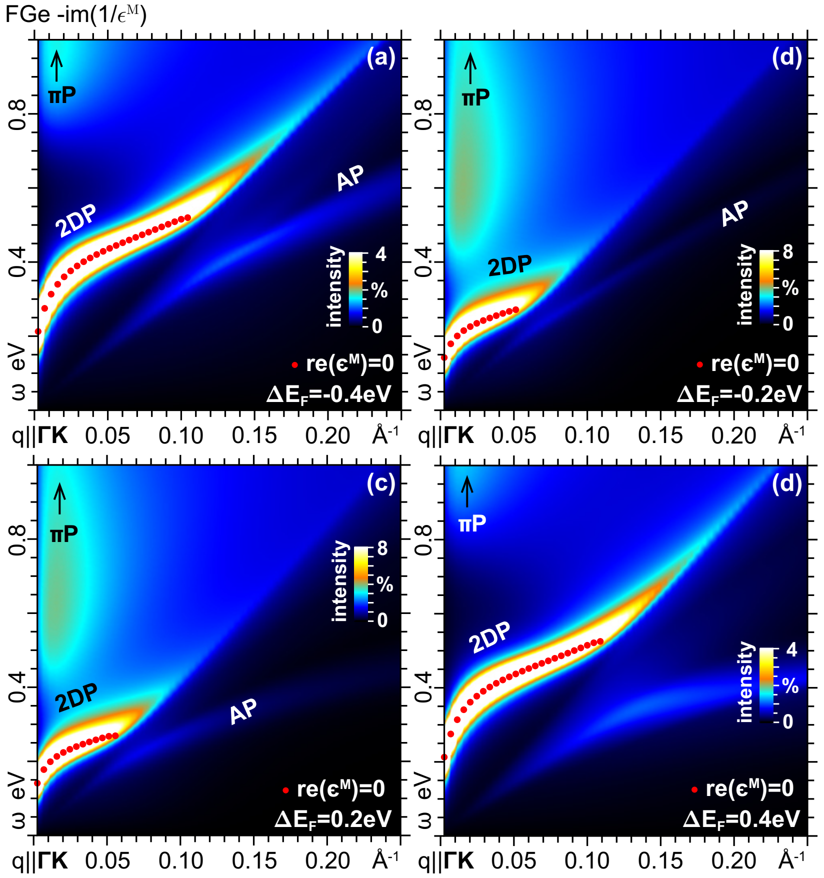

Figure S10
Loss function of FGe under the same extrinsic conditions of Fig. S9, for momentum transfers $\mathbf{q}||\Gamma\mathrm{K}$. The panel-by-panel comparison with Fig. S9 shows an AP mode, which is absent in the $\mathbf{q}||\Gamma\mathrm{M}$-case. The same feature has also been found in graphene and silicene[37,38]. All other settings are as in Fig. S9.

SIV. DIELECTRIC PROPERTIES OF QFGE ON ALN AND MOS_2

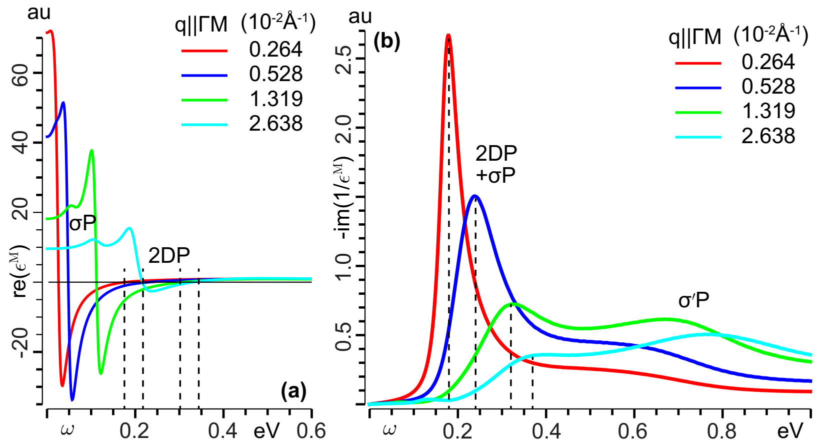

Figure S11
(a) Real permittivity and (b) loss function ($\propto$-im($1/\epsilon^{\mathrm{M}}$)) of intrinsic QFGe on MoS_2 for ω<1 eV and some $\mathbf{q}\|\Gamma$M, with $|\mathbf{q}|$<2.7 Å^{-1}. (a) and (b) respectively provide complementary information to Fig.4 (f) and 4 (c). The undamped features of the 2DP mode are attested by the double change in re(ϵ^{M}), with the highest zero of re(ϵ^{M}) being preceded by a plasmon resonance peak in -im($1/\epsilon^{\mathrm{M}}$) for each specific momentum transfer (dashed lines). Also observable are the oscillations in re(ϵ^{M}) below the 2DP resonance, which may indicate that the σP peak lies before the 2DP peak in $-$im($1/\epsilon^{\mathrm{M}}$).

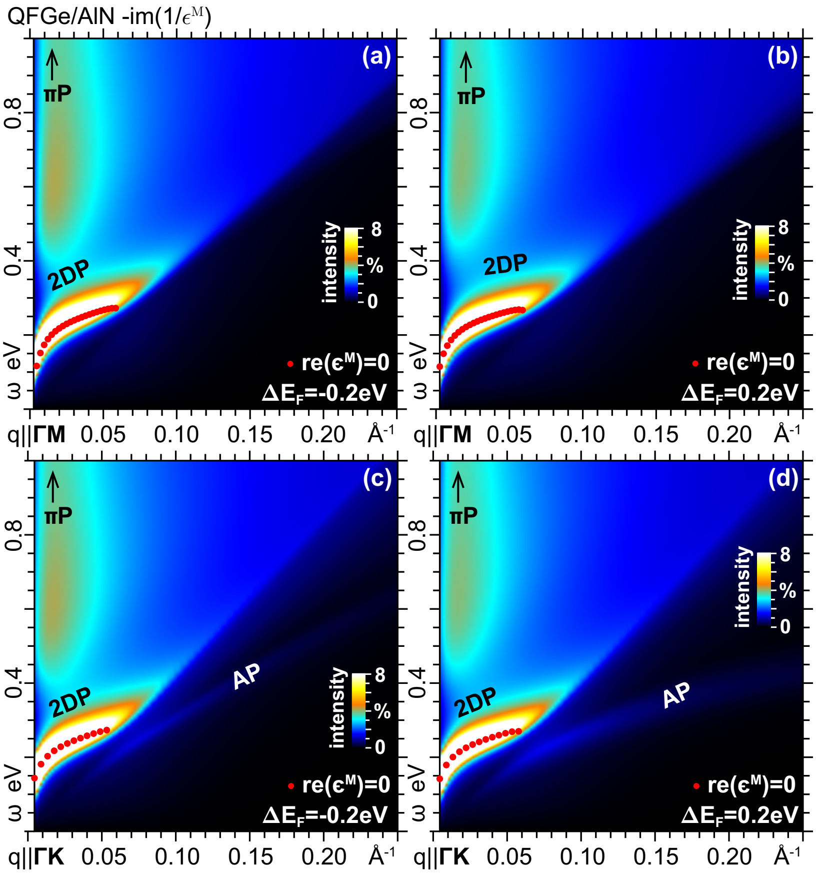

Figure S12
Loss function of QFGe on AlN for the extrinsic conditions ΔE_{F}=$\pm$0.2 and momentum transfers (a), (b) $\mathbf{q}\|\Gamma$M, (c), (d) $\mathbf{q}\|\Gamma$K, which lead to a scenario typical of 2DDMs with group IV atomic elements, including FGe in the LDA geometry (see Figs. S9, S10 above). This oscillation of the massless charge carriers can be individually tuned by changing the Fermi level position in the range where the σ-like or σ^*-like states are left inert, namely $-0.31\leq\Delta E_{\mathrm{F}}\leq1.00$

eV, as also suggested by the associated band dispersions (see Fig. 1b of the main text). The dominant 2DP propagates mostly undamped at MIR to NIR energies, and a small AP is activated by an applied momentum $\mathbf{q}\|\Gamma K$.

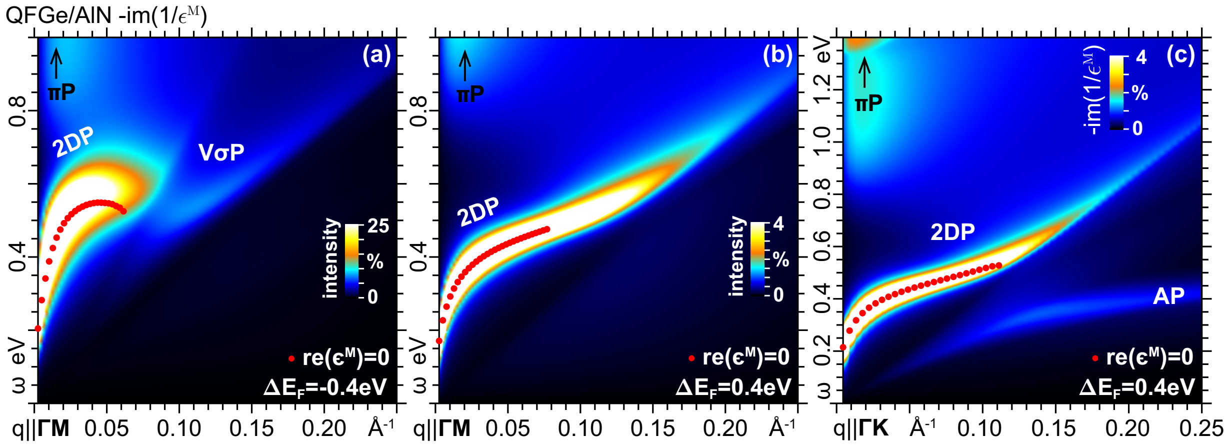

Figure S13
Loss function of QFGe on AlN for the extrinsic conditions (a) ΔE_{F}=-0.4 eV, (b), (c) ΔE_{F}=0.4 eV, and momentum transfers (a), (b) $\mathbf{q}\|\Gamma M$, (c) $\mathbf{q}\|\Gamma K$. (a) ΔE_{F}=-0.4 eV describes the same peculiar situation of Fig. 7(b), with $\mathbf{q}\|\Gamma M$ in spite of $\mathbf{q}\|\Gamma K$, as attested by the absence or presence of the AP. In both the $\mathbf{q}\|\Gamma K$ and $\mathbf{q}\|\Gamma M$ cases, the Fermi level is driven around the top of the σ states, which generates a V-shaped massive mode on the same energy range as the 2DP, but shifted in momentum space. The intensity color scale is the same as in Fig. 7(b). (b), (c) ΔE_{F}=0.4 eV offers a similar picture as Fig. S12 above, with a dominant 2DP propagating mostly undamped at NIR to MIR energies, and the AP mode being (b) deactivated by the applied momentum $\mathbf{q}\|\Gamma M$, and (c) activated y $\mathbf{q}\|\Gamma K$.

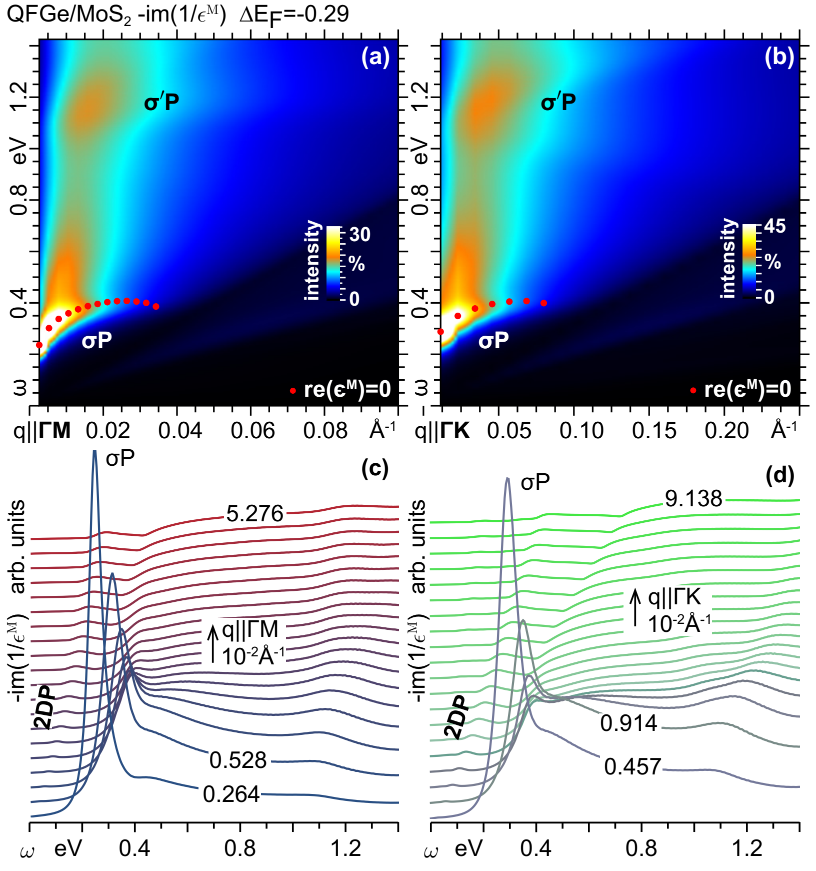

Figure S14
Loss function of QFGe on MoS_2 for the extrinsic condition $\Delta E_{\mathrm{F}} = -0.29$ eV that drives the Fermi level at the Dirac cone vertex. A complementary view of Fig. 5(c) and Fig. 6a of the main text is offered, which provides a complete analysis of the intraband σP and σ'P for $\mathbf{q}\|\Gamma K$ and $\mathbf{q}\|\Gamma M$. In the shifted loss spectra of (c) and (d), a tiny 2DP is barely

visible at energies similar to the corresponding excitation in intrinsic FGe [Fig. 5(a)] and QFGe on AlN [Fig. 5(b)].

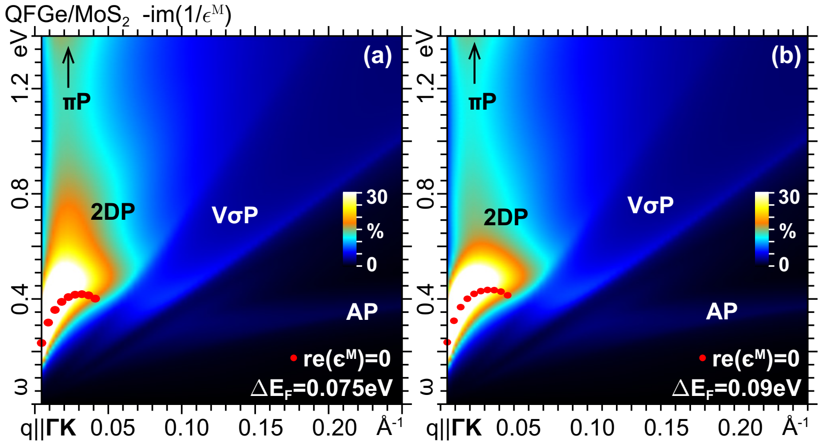

Figure S15
Loss function of QFGe on MoS_2 for (a) $\Delta E_F = 0.075\,eV$, (b) $\Delta E_F = 0.09\,eV$ and $\mathbf{q}||\Gamma K$. As in Fig. 7(a), (b) of the main text, the Fermi level is driven close to the top of the σ-like band, which causes the appearance of a V-shaped massive plasmon disjoint from the 2DP and shifted in momentum space. All other settings are as in Figs. S10, S12, S13, S14(a),(b).

SV. PW-DFT CALCULATIONS WITHIN THE LDA AND GGA

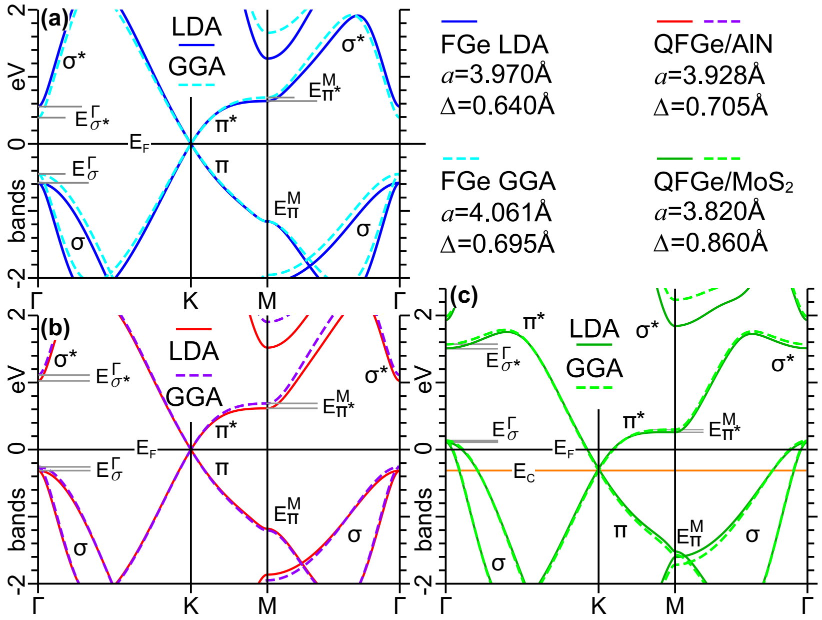

Figure S16
LDA *vs* GGA bands of (a) FGe, (b) QFGe on AlN, and (c) QFGe on MoS_2. As reported in the legend, the replacement of the LDA with the GGA causes an expansion of the FGe lattice, whereas the defining parameters of the two QFGe lattices are fixed by the experimental conditions[23,24]. In all three monolayers, the two highest bands below and the lowest band above E_C show almost identical profiles in an energy windows of $\sim$2.5 eV around E_F, apart from the following exceptions around the Γ and M points: (a) in FGe, the significant discrepancies in LDA and GGA geometries cause a decrease of the GGA band gap at Γ by 0.30 eV. Additionally, the GGA unoccupied VHS energy E^{π}_{M} is shifted up by 0.05 eV away from E_F; (b) in QFGe on AlN, the occupied and unoccupied σ energies at Γ, respectively differ by 0.09 eV and 0.06 eV, while the GGA unoccupied VHS energy E^{π}_{M} is shifted up by 0.07 eV away from E_F; (c) in QFGe on MoS_2, the occupied and unoccupied σ energies at Γ, respectively differ by 0.02 eV and 0.06 eV, while the GGA unoccupied VHS energy E^{π}_{M} is shifted up by 0.04 eV away from E_F. As a result, the GGA and LDA dielectric responses of the QFGe monolayers have almost undetectable differences for probing frequencies $\omega \lesssim 2.5\,eV$.
